# HNF4A mitigates sepsis-associated lung injury by upregulating NCOA2/GR/STAB1 axis and promoting macrophage polarization towards M2 phenotype

**DOI:** 10.1038/s41419-025-07452-z

**Published:** 2025-02-21

**Authors:** Yu-Hang Yang, Ri Wen, Xin-Mei Huang, Tao Zhang, Ni Yang, Chun-Feng Liu, Tie-Ning Zhang

**Affiliations:** 1 Department of Pediatrics, PICU, Shengjing Hospital of China Medical University, Shenyang, China; 2 Department of Endocrinology, Shanghai Fifth People’s Hospital, Fudan University, Shanghai, China

**Keywords:** Bacterial infection, Inflammatory diseases

## Abstract

Sepsis can trigger systemic inflammation and lead to detrimental effects on several organs, with particular emphasis on the lungs. In sepsis-associated lung injury, macrophages assume a pivotal role, as their overactivation could facilitate the secretion of inflammatory factors and the imbalance of polarization. Hepatocyte nuclear factor 4 alpha (HNF4A) has been reported its potential involvement in the regulation of inflammatory response and macrophage polarization. This study discusses the role and mechanism of HNF4A in sepsis-induced lung damage. HNF4A exhibits a decrease in expression by analyzing the differentially expressed genes in the lungs of septic mice from the Gene Expression Omnibus dataset GSE15379. Then, we established a mouse sepsis model through a cecal ligation and puncture method and observed that the expression of HNF4A was reduced in both lung tissues and alveolar macrophages. To evaluate the function of HNF4A, we overexpressed HNF4A mediated by adenovirus vectors, which were injected into mice. We found that HNF4A overexpression resulted in a higher survival rate in septic mice and an amelioration of pulmonary damage. Meanwhile, HNF4A overexpression mitigated the infiltration of inflammatory cells and impeded the M1 polarization but facilitated the M2 polarization of macrophages in the lung tissues or the alveolar lavage fluid. In vitro, we treated bone marrow-derived macrophages with interleukin-4. Consistent results were obtained that HNF4A overexpression promoted the M2 polarization of macrophages. Mechanistically, we found that HNF4A transcriptionally regulate the expression of nuclear receptor coactivator 2 (NCOA2) through binding to its promoter region. NCOA2 interacted with glucocorticoid receptor (GR). Stabilin 1 (STAB1) was selected as a possible target by transcriptome sequencing analysis. Functional experiments confirmed STAB1 as a downstream target of the HNF4A/NCOA2/GR axis. Overall, this research investigated the potential impact of HNF4A on pulmonary injury in sepsis. It is suggested that one of the regulatory mechanisms involved in this association may be the NCOA2/GR/STAB1 axis.

## Introduction

Sepsis is a systemic inflammatory response syndrome triggered by infection that can result in multiple organ dysfunction [1]. It is characterized by symptoms such as fever, tachycardia, dyspnea, and imbalanced white blood cell counts [2]. The lung is the predominant organ affected by sepsis [3], and the mortality rate associated with acute lung damage (ALI) resulting from sepsis can reach up to 40%, surpassing the fatality rates contributed by alternative sources of ALI [4]. Macrophages play a key role in the pathogenesis of pulmonary damage in sepsis [5]. Normally, macrophages, as an important part of the immune system, are responsible for the elimination of infections and apoptotic cells, as well as the preservation of tissue homeostasis [6]. However, these cells become excessively active during the progression of sepsis, resulting in an exaggerated inflammatory response, an imbalance in polarization, and dysfunction in endophagocytosis, all of which contribute to the acceleration of the damage response [7].

Macrophages can transition between two primary phenotypes, namely M1 (classical activation, pro-inflammatory) and M2 (replacement activation, anti-inflammatory), in response to signals received from the microenvironment to maintain tissue homeostasis [8]. However, in the context of sepsis-related danger signals such as pathogen-associated molecular patterns (PAMPs) and injury-associated molecular patterns (DAMPs), the balance tends to support M1 polarization [9]. The M1-type macrophages further promote the production and release of pro-inflammatory cytokines and reactive oxygen species (ROS), resulting in heightened lung inflammation and oxidative stress and inducing lung injury [10]. In recent years, intervention of macrophage polarization with pharmacological or biological agents to promote the transformation of M1 to M2 has emerged as a promising approach for the management of pulmonary injury linked to sepsis [11–13]. However, the molecular targets of sepsis-associated lung injury remain largely unelucidated, which has hindered subsequent development.

Hepatocyte nuclear factor 4 alpha (HNF4A) is mainly known for its role in the regulation of hepatic metabolic pathways [14]. It is reported that HNF4A can alleviate acute liver injury generated by acetaminophen [15] and liver fibrosis caused by toxins and cholestasis [16]. Recently, some studies have suggested that HNF4A may also play a role in the indirect modulation of inflammatory responses [17, 18]. Notably, in Yang et al.’ paper, they proved that HNF4A can exert a protective influence against liver fibrosis through its regulation of liver macrophage polarization [16]. By analyzing the dataset GSE15379 from Gene Expression Omnibus (GEO) database, we found a notable decrease in the expression of HNF4A in the lung tissue of septic mice with a cecal ligation and puncture (CLP) method, as compared to the control group (log2FC = −1.26; *p* < 0.01). Therefore, it is postulated that the down-regulation of HNF4A may potentially play a role in the pathogenesis of sepsis-associated pulmonary damage by modulating the phenotype transition of macrophages.

The glucocorticoid receptor (GR) is a cellular protein that exhibits binding affinity towards glucocorticoids such as cortisol, hence exerting an influence on the regulation of gene expression [19]. Once the hormone establishes a binding interaction with GR, the resultant complex is capable of translocating into the nucleus and binding to a specific DNA sequence known as the glucocorticoid response element (GRE), thereby acting as a transcription factor [20]. This transcription factor possesses its ability to either start or impede the transcription of specific genes, thereby regulating a variety of cellular processes such ad inflammatory response, immunological function, and metabolism [21]. Nuclear receptor coactivator 2 (NCOA2) is a nuclear receptor coactivator of GR [22]. It can directly bind to the GR monomers or homologous dimers to participate in the regulation of GR-mediated gene transcription [23]. Moreover, deletion of NCOA2 in obese mice elevated the expression of M1-type factors but reduced the M2-type factors in macrophages of the adipose tissues [24]. The above literature comprehensively suggests that NCOA2 and GR are significant contributors to the survival and anti-inflammatory M2 phenotype of macrophages in sepsis.

Intriguingly, the JASPAR database indicates binding sites between HNF4A and the NCOA2 promoter. Therefore, we hypothesize that HNF4A may exert a regulatory role in the sepsis-associated lung injury by influencing NCOA2/GR-mediated gene transcription. In this work, we conducted in vivo and in vitro experiments to investigate the role of HNF4A. In addition, we employed transcriptome sequencing method to explore the gene regulation mediated by HNF4A/NCOA2/GR axis.

## Results

### Transcriptomic analysis of lung tissues from CLP-induced septic mice and the sham mice in GSE15379

To investigate the molecular regulatory mechanisms associated with sepsis-induced ALI, we analyzed the profiles of transcriptomics changes in lung tissues of CLP-induced septic mice compared with the sham mice from GSE15379. The heatmap showed the total differentially expressed genes (DEGs) (∣log2FoldChange∣> 1, and −log10 (*p*-value) > 2.5) (Supplementary Figure 1A). Next, we performed the GO analysis for DEGs. The enrichment results of biological process (BP) showed that the most of DEGs are related to inflammation. The enrichment of molecular function (MF) identified that the majority of DEGs are related to transcription (Supplementary Figure 1B). We focused on the top 5 GO terms associated with transcription factors from DEGs. By Venn diagram analysis, we found that 10 overlapping DEGs (CEBP, FOXC1, CEBPD, HNF4A, JUNB, STAT3, FOS, TBX3, CREB1, TRP53) associated with the top 5 GO-MF terms (Supplementary Figure 1C-D). Notably, HNF4A is a transcription factor in DEGs. Furthermore, the HNF4A expression is down-regulated in lung tissues of CLP-induce septic mice compared with the sham mice in GSE 15379. Currently, the effect of HNF4A on ALI has not been reported, which is worthy of further investigation.

### The expression of HNF4A is down-regulated during sepsis-induced ALI

**Fig. 1:**
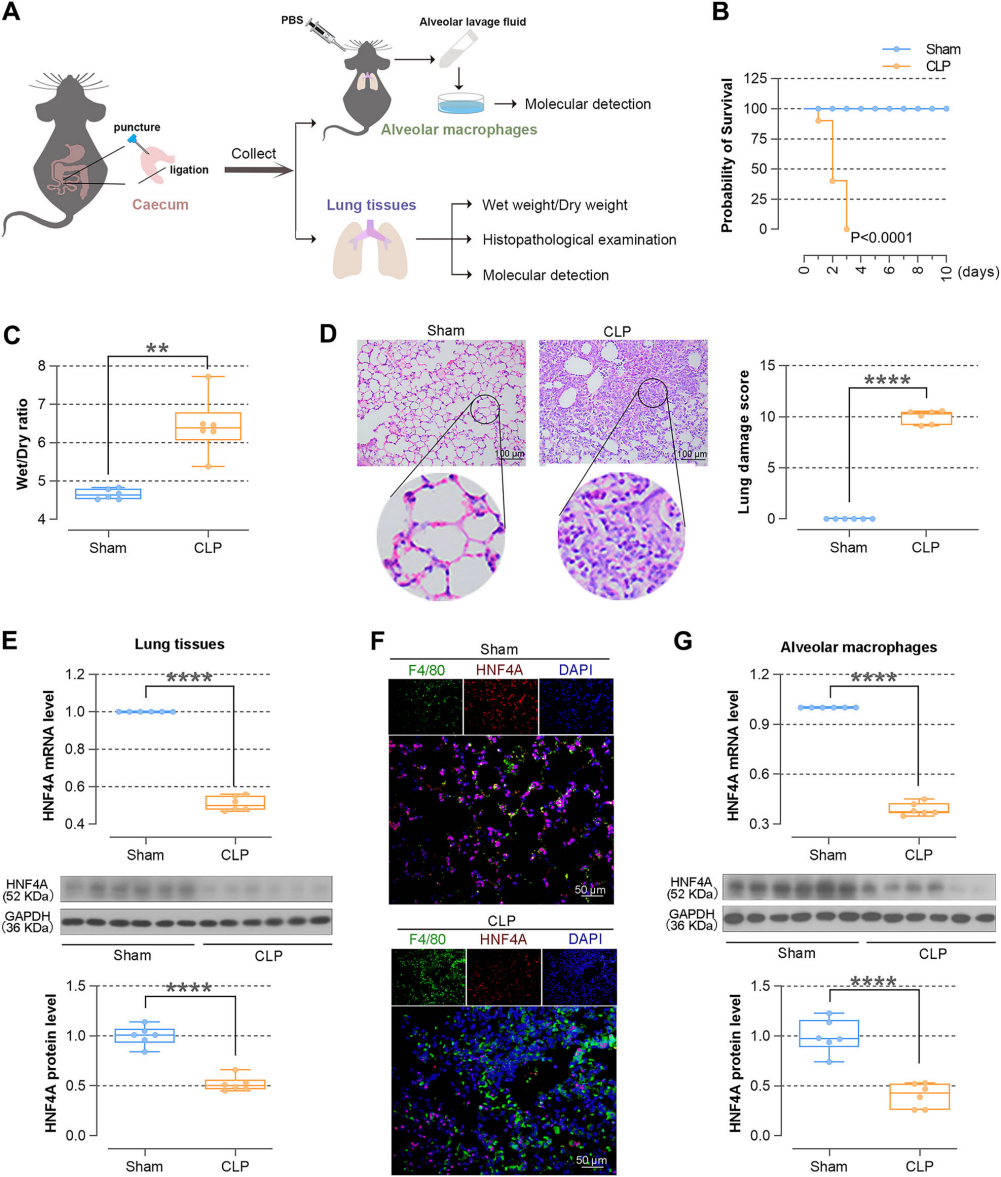
The expression of HNF4A in lung tissues of septic mice is down-regulated. **A** Scheme to establish the mouse model of sepsis by cecal ligation and puncture (CLP). Schematics are obtained from SciDraw. Mouse: 10.5281/zenodo.3926105; Petrified Dish:10.5281/zenodo.6808892; Falcon Tube: 10.5281/zenodo.4421153; Syringe: 10.5281/zenodo.4152947. **B** The survival probability of mice is measured. **C** Comparison of wet/dry weight ratio of lung tissues of mice in sham surgery mice and CLP mice. **D** Hematoxylin and eosin (H&E) staining (×200) of lung tissues and lung damage scores in sham surgery mice and CLP mice. **E** The expression of HNF4A is assayed by real-time PCR and western blot (WB) in lung tissues. **F** Immunofluorescence (IF) staining (×400) of F4/80 and HNF4A shows the expression of HNF4A in alveolar macrophages. **G** The expression of HNF4A is assayed by real-time PCR and WB in alveolar macrophages. The data are presented as mean ± SD. **P < 0.01, ****P < 0.0001.

The mouse model of sepsis was constructed by CLP induction to verify the situation of lung injury and changes in HNF4A expression (Fig. 1A). As shown in Fig. 1B, the mortality rate of CLP-induced mice was higher than the sham mice. The lung tissues of CLP-induced mice showed a higher Wet/Dry ratio, indicating severe edema of lung issues after CLP-induced sepsis (Fig. 1B). The infiltration of inflammatory cells was obviously observed by HE staining in lung tissues of CLP-induced mice, and the score of lung damage also increased (Fig. 1D). Next, the alteration of HNF4A expression was determined. We found that the expression levels of HNF4A were down-regulated in lung tissues of CLP-induced mice (Fig. 1E). Alveolar macrophages were isolated from alveolar lavage fluid. The levels of HNF4A were down-regulated in alveolar macrophages, which were also corroborated by IF staining (Fig. 1F-1G). Collectively, these results suggested that lung tissues are severely damaged after CLP-induced sepsis, mainly manifested as pulmonary edema and inflammatory cell infiltration, and HNF4A levels are down-regulated.

### Overexpressing HNF4A alleviates lung injury and suppresses inflammation in septic mice

**Fig. 2:**
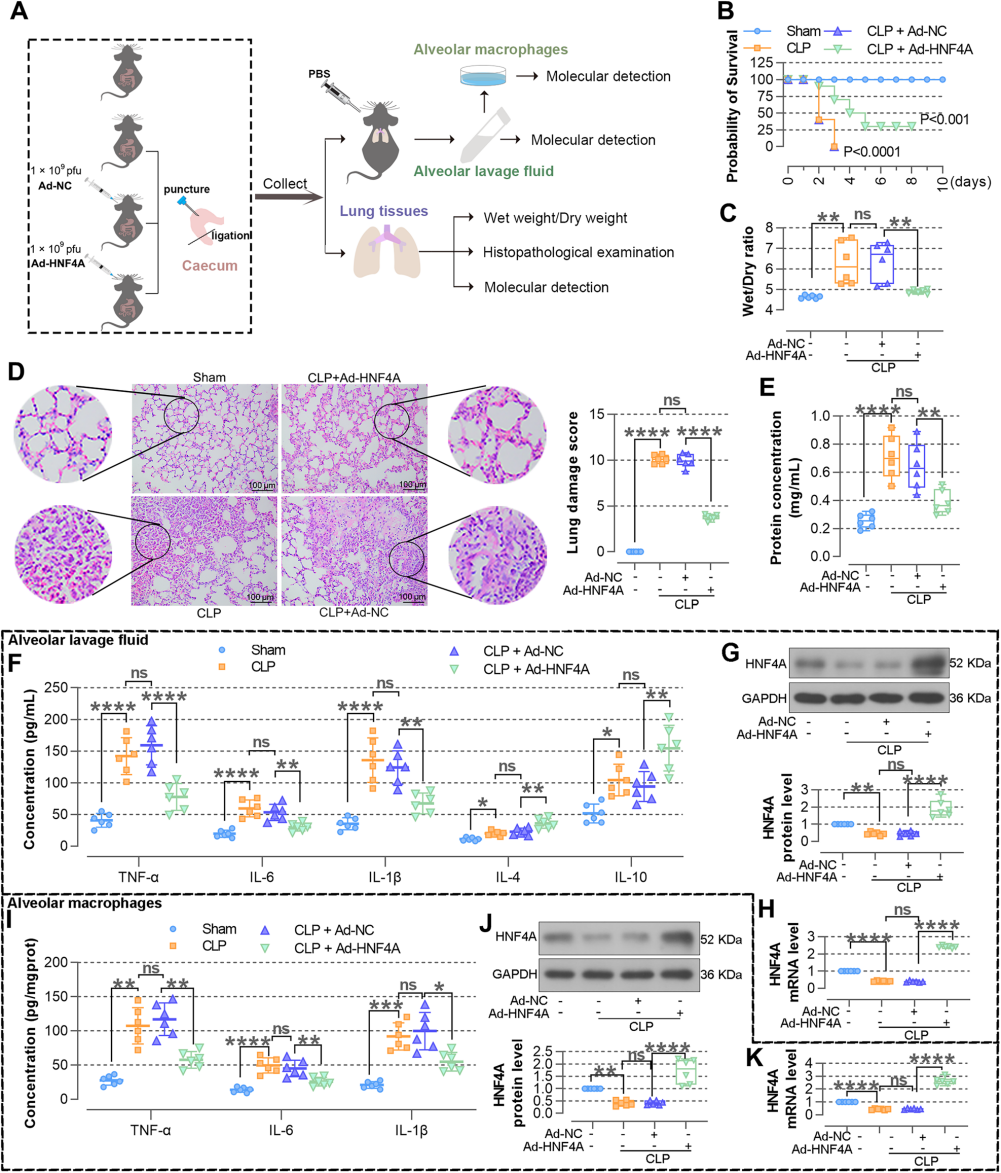
Overexpressing HNF4A alleviates lung injury and suppresses inflammation in septic mice. **A** Mice are instilled with 1 × 10^9^ pfu of adenoviruses overexpressing HNF4A (Ad-HNF4A) or negative controls (Ad-NC), followed by being induced with sepsis by CLP. Schematics are obtained from SciDraw. Mouse: 10.5281/zenodo.3926105; Petrified Dish: 10.5281/zenodo.6808892; Falcon Tube: 10.5281/zenodo.4421153; Syringe: 10.5281/zenodo.4152947. **B** The survival probability of mice is detected. **C** Comparison of wet/dry weight ratio of lung tissues of mice. **D** H&E staining (×200) of lung tissues and lung damage scores. **E** The concentration of total protein in alveolar lavage fluid is measured. **F** Inflammatory factors (TNF-α, IL-1β, IL-6, IL-4, and IL-10) representative of pro-inflammatory macrophages and anti-inflammatory macrophages are detected in the alveolar lavage fluid. **G** The expression of HNF4A is measured by WB in lung tissues. **H** The expression of HNF4A is measured by real-time PCR in lung tissues. **I** Inflammatory factors (TNF-α, IL-1β, and IL-6) representative of pro-inflammatory macrophages are detected in macrophages from alveolar lavage fluid. **J** The expression of HNF4A is measured by WB in macrophages from alveolar lavage fluid. **K** The expression of HNF4A is measured by real-time PCR in macrophages from alveolar lavage fluid. The data are presented as mean ± SD. *P < 0.05, **P < 0.01, ***P < 0.001, ****P < 0.0001.

To clarify the role of HNF4A in ALI, we overexpressed HNF4A mediated by adenovirus prior to CLP induction, as confirmed by the Real-time PCR and western blot (WB). (Fig. 2A, G and H). On day 3, the probability of survival was 0% for the septic mice instilled with Ad-NC, while the probability of survival for the septic mice instilled with Ad-HNF4A was 70% (Fig. 2B). Pulmonary edema was improved by HNF4A overexpression in CLP-induced mice, as evidenced by decreased lung Wet/Dry ratio (Fig. 2C). Compared with the septic mice instilled with Ad-NC, histological results showed the decreased infiltration of inflammatory cells in lung tissues of the septic mice instilled with Ad-HNF4A (Fig. 2D). Meanwhile, the concentration of total protein in alveolar lavage fluid was decreased by HNF4A overexpression (Fig. 2E). We detected the levels of M1 and M2 inflammatory cytokines in the alveolar lavage fluid. As shown in Fig. 2F, the levels of IL-4 and IL-10 were increased by HNF4A overexpression, while the levels of TNF-α, IL-1β, and IL-6 were decreased. Consistently, we found that HNF4A overexpression decreases levels of TNF-α, IL-1β, and IL-6 in alveolar macrophages (Fig. 2I–K). Taken together, these findings implied that HNF4A overexpression suppresses inflammation and improves ALI in CLP-induced mice.

### Overexpressing HNF4A promotes the polarization of macrophages towards M2 phenotype and upregulates the expression of NCOA2 in septic mice

**Fig. 3:**
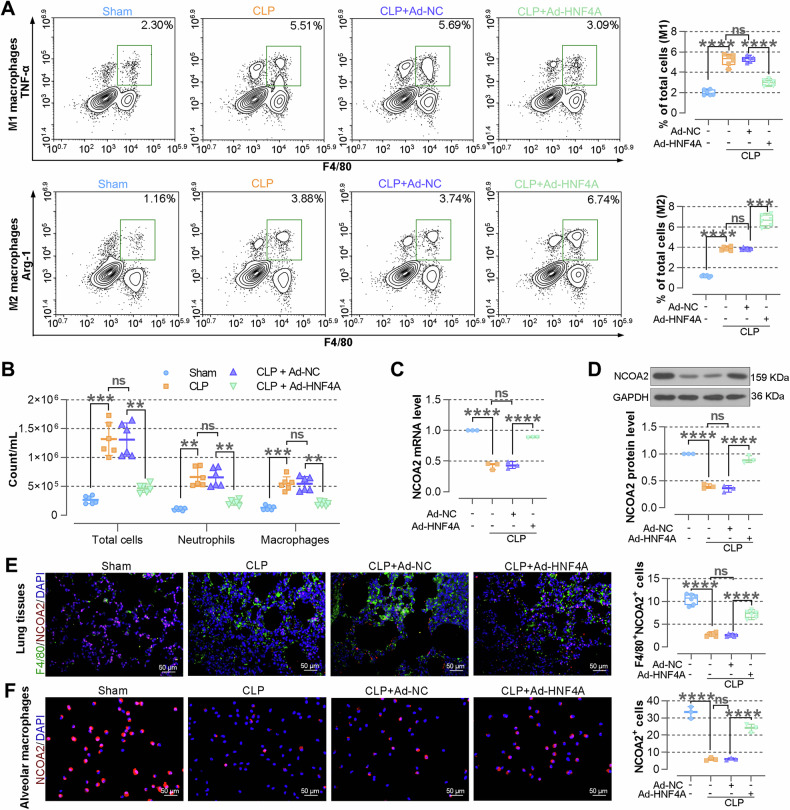
Overexpressing HNF4A promotes the polarization of macrophages towards M2 phenotype and upregulates the expression of NCOA2 in septic mice. **A** The expressions of TNF-α and Arg-1 in alveolar lavage fluid are detected by flow cytometry. pro-inflammatory macrophages are F4/80^+^ TNF-α^+^ cells, whereas anti-inflammatory macrophages are F4/80^+^ Arg-1^+^ cells. **B** Total cells, neutrophils, and macrophages in the alveolar lavage fluid are counted by Wright-Giemsa staining. **C** The expression of NCOA2 is assayed by real-time PCR in alveolar macrophages. **D** The expression of NCOA2 is assayed by WB in alveolar macrophages. **E** The expression of NCOA2 in lung tissues is observed by IF staining (×400) of F4/80 and NCOA2. **F** The expression of NCOA2 in alveolar macrophages is observed by IF staining (×400). The data are presented as mean ± SD. *P < 0.05, **P < 0.01, ***P < 0.001, ****P < 0.0001.

To explore the impact of HNF4A on the macrophage polarization, TNF-α and Arg-1 expressions on macrophages (F4/80-positive cells) were measured by flow cytometry. Compared with septic mice instilled with Ad-NC, HNF4A overexpression resulted in an increase in macrophages towards M2 phenotype and a decrease in macrophages towards M1 phenotype (Fig. 3A). As shown in Fig. 3B, the number of total leukocytes, neutrophils, and macrophages remarkably reduced in the alveolar lavage fluid of HNF4A-overexpressed mice. Moreover, the overexpression of HNF4A resulted in an increase in the expressions of NCOA2 in alveolar macrophages of septic mice. (Fig. 3C, D). Immunofluorescence assays (Fig. 3E, F) were performed to further verify the results of real-time PCR and WB. In sum, these findings indicated HNF4A overexpression promotes the polarization of alveolar macrophages towards M2 phenotype and up-regulates the levels of NCOA2.

### Overexpressing HNF4A promotes the polarization of BMDMs towards M2 phenotype and HNF4A binds to the NCOA2 promoter

**Fig. 4:**
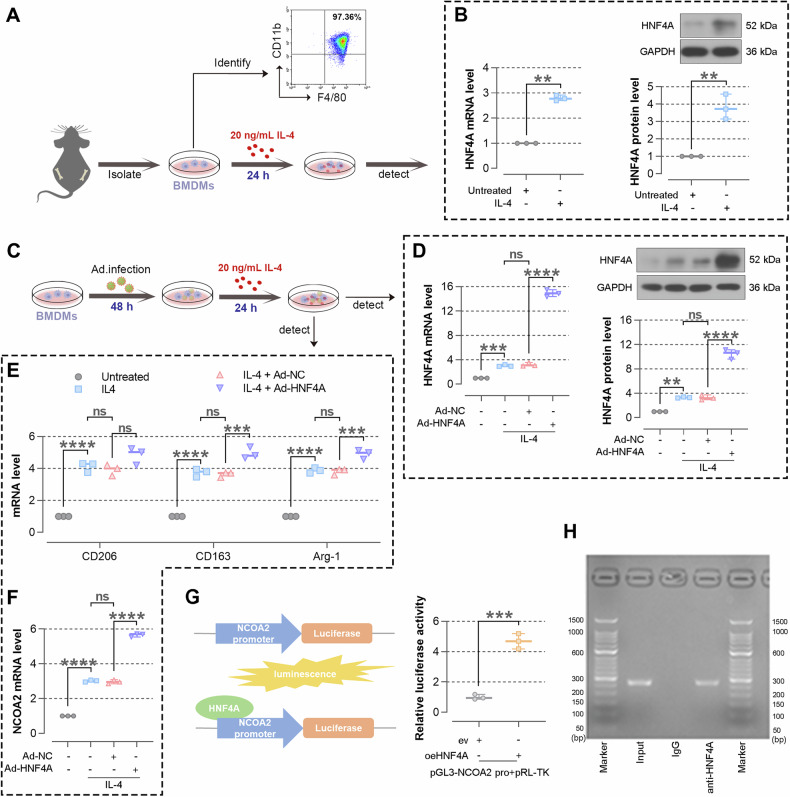
Overexpressing HNF4A promotes the polarization of BMDMs towards M2 phenotype and HNF4A binds to the NCOA2 promoter. **A** Isolation and culture of mouse bone BMDMs. M2-type polarization is induced by treating BMDMs with IL-4 for 24 h. Schematics are obtained from SciDraw. Mouse: 10.5281/zenodo.3926105; Adenovirus: doi.org/10.5281/zenodo.3926233. **B** The expression of HNF4A in cells is detected by real-time PCR and WB. **C** Ad-HNF4A infected BMDMs for 48 h, followed by inducing M2 polarization with IL-4 for 24 h. **D** The expression of HNF4A in BMDMs is detected by real-time PCR and WB. **E** The expressions of CD206, CD163, and Arg-1 are detected by real-time PCR in BMDMs. **F** The expression of NCOA2 is measured by real-time PCR in BMDMs. **G** Dual luciferase reporter assays are performed with 293 T cells co-transfected with pGL3-basic luciferase reporter containing NCOA2 promoter (pGL3-NCOA2 pro) and the HNF4A overexpression or empty vector. Luciferase activities are measured and normalized to the *Renilla* activity. **H** ChIP assay is performed to show that HNF4A binds to the NCOA2 promoter. The data are presented as mean ± SD. **P < 0.01, ***P < 0.001, ****P < 0.0001.

We isolated BMDMs from the bone marrow of C57BL/6J mice and identified the cells by flow cytometry (Fig. 4A). Subsequently, BMDMs were treated with IL-4 for 24 h to induce polarization towards M2 phenotype and treated with LPS and IFN-γ to induce polarization towards M1 phenotype. The results of WB and Real-time PCR showed that HNF4A levels are up-regulated (Fig. 4B). In order to investigate the impacts of HNF4A on the polarization of BMDMs, BMDMs were infected with Ad-HNF4A to overexpress HNF4A and then induced polarization towards M2 phenotype (Fig. 4C) and. As shown in Fig. 4D, HNF4A was overexpressed in IL-4-induced BMDMs, as evidenced by WB and real-time PCR (Fig. 4D). The expression levels of CD206, CD163, and Arg-1 (markers of M2 phenotype) were up-regulated after HNF4A overexpression, indicating that HNF4A promotes the polarization of BMDMs towards M2 phenotype (Fig. 4E). In contrast, the expression levels of iNOS and MCHII (markers of M1 phenotype) were down-regulated after HNF4A overexpression in LPS + IFN-γ-induced BMDMs (Supplementary Fig. 2A, B). The levels of TNF-α, IL-1β, and IL-6 were also down-regulated, suggesting that HNF4A suppressed the polarization of BMDMs towards M1 phenotype (Supplementary Fig. 2C). Furthermore, we found that the expression of NCOA2 is also increased (Fig. 4F). To confirm whether HNF4A modulates the NCOA2 expression by binding to the NCOA2 promoter, we performed the dual luciferase reporter assay to assess the activity of pGL3-NCOA2 promoter luciferase reporter. The result showed that HNF4A contributes to the activation of pGL3-NCOA2 promoter luciferase activity (Fig. 4G). To further verify whether HNF4A binds to the NCOA2 promoter directly, we employed an antibody against HNF4A for immunoprecipitation, followed by PCR analysis in IL-4-induced BMDMs. The results showed that immunoprecipitated products using IgG did not contain the NCOA2 promoter. In contrast, the NCOA2 promoter sequence was detected in the immunoprecipitation product using the HNF4A antibody, suggesting that HNF4A binds to the NCOA2 promoter directly (Fig. 4H). Taken together, these results indicated that HNF4A is able to bind to the NCOA2 promoter, promotes the polarization of BMDMs towards M2 phenotype and suppresses the polarization of BMDMs towards M1 phenotype.

### HNF4A affects the polarization of macrophages via NCOA2/GR pathway

**Fig. 5:**
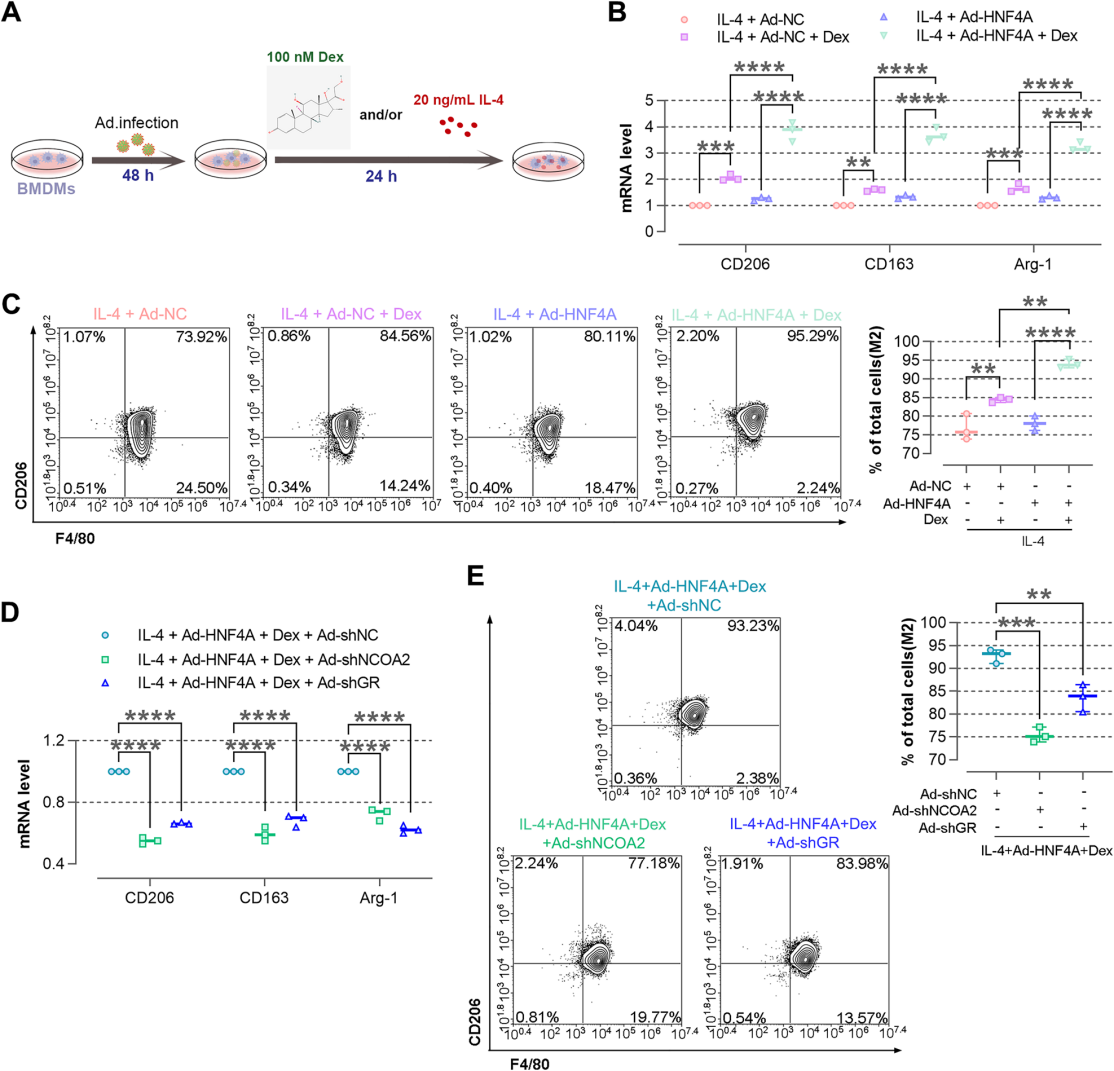
HNF4A affects the polarization of macrophages via NCOA2/GR pathway. **A** After being infected with Ad-HNF4A or Ad-NC for 48 h, IL-4 and/or 100 nM Dex is added. Schematics are drawn via SciDraw. Adenovirus: doi.org/10.5281/zenodo.3926233. The structure formula of Dex is from Pubchem. **B** The expressions of CD206, CD163, and Arg-1 are detected by real-time PCR in BMDMs. **C** The expression of CD206 in BMDMs is detected by flow cytometry. **D** The expressions of CD206, CD163, and Arg-1 are detected by real-time PCR in BMDMs. **E** The expression of CD206 in BMDMs is detected by flow cytometry. The data are presented as mean ± SD. **P < 0.01, ***P < 0.001, ****P < 0.0001.

To investigate whether HNF4A affects polarization of BMDMs in the activated state of GR pathway, BMDMs were infected with Ad-HNF4A for 48 h, and Dex was added to activate the GR pathway before inducing polarization (Fig. 5A). After the addition of Dex, HNF4A overexpression up-regulated the levels of CD206, CD163, and Arg-1, indicating that HNF4A overexpression significantly promotes BMDMs polarization towards M2 phenotype in the activated state of GR pathway (Fig. 5B, C). On the contrary, HNF4A overexpression down-regulated the levels of iNOS, MCHII, TNF-α, IL-1β, and IL-6, indicating that HNF4A overexpression significantly suppresses BMDMs polarization towards M1 phenotype in the activated state of GR pathway (Supplementary Fig. 2D-G). Subsequently, we further explored the regulatory mechanism by knocking down NCOA2 or GR. Under the conditions of adding Dex and overexpressing HNF4A, we found that knocking down GR inhibits BMDMs polarization towards M2 phenotype and promotes BMDMs polarization towards M1 phenotype. Consistently, knocking down NCOA2 also inhibits BMDMs polarization towards M2 phenotype and promotes polarization towards M1 phenotype (Fig. 5D, E, Supplementary Fig. 2H–K). Taken collectively, HNF4A significantly promoted the polarization of BMDMs towards M2 phenotype and inhibited the polarization of BMDMs towards M1 phenotype via the NCOA2/GR pathway.

### mRNA-seq whole transcriptome profiling in NCOA2-knockdowned BMDMs treated with Ad-HNF4A, IL-4 and Dex

**Fig. 6:**
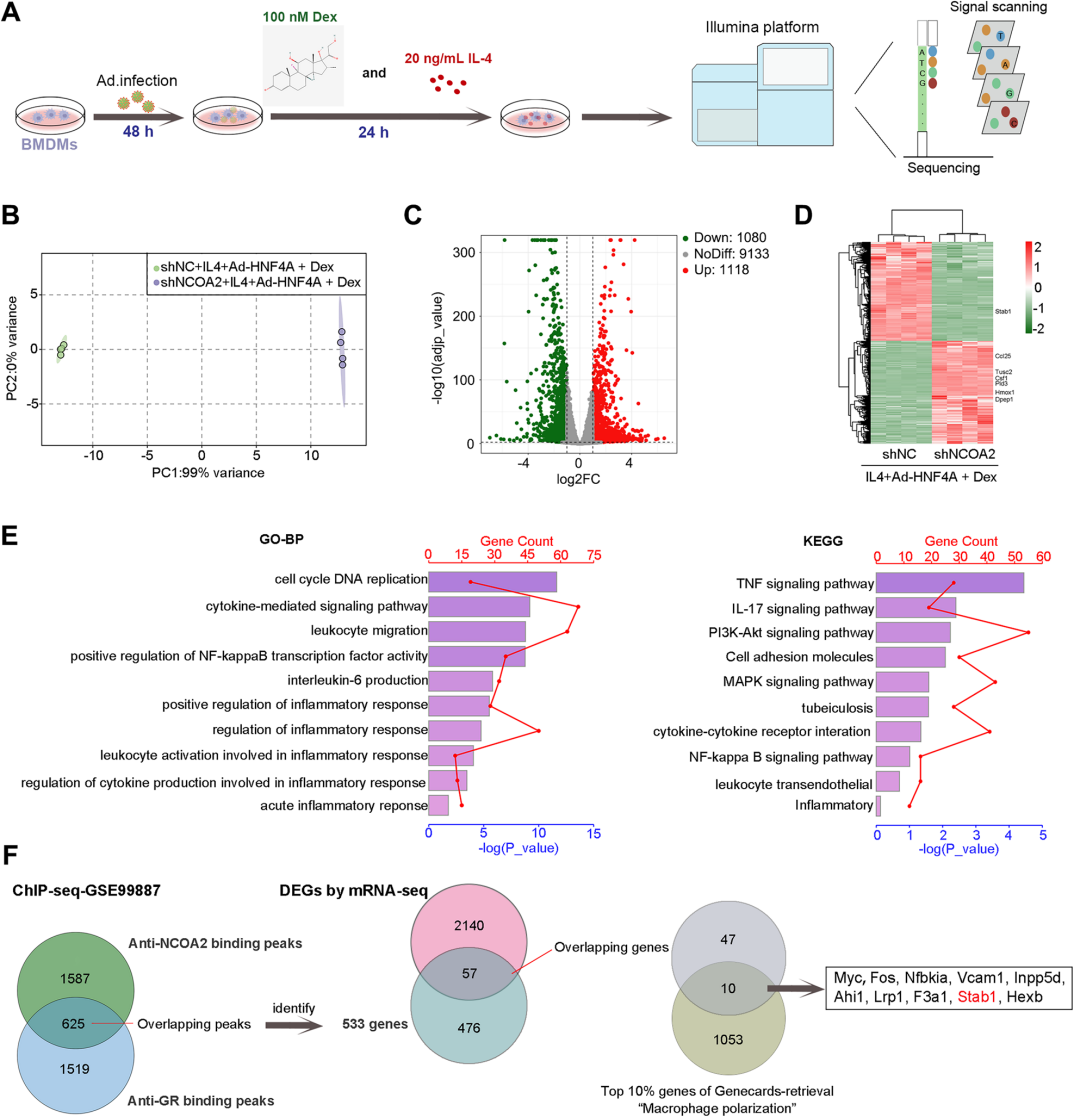
mRNA-seq profiling in NCOA2-knockdown BMDMs treated with Ad-HNF4A, IL-4 and Dex. **A** BMDMs infected with Ad-HNF4A are infected with Ad-shNCOA2 or Ad-shNC for 48 h, and then cells are cultured with IL-4 and Dex for 24 h followed by transcriptome analysis. **B** Principal-component analysis (PCA) analysis. **C** Volcano plot of differentially expresses genes (DEGs). **D** The heatmap of DEGs. **E** GO and KEGG enrichment analysis of DEGs. **F** Venn diagram shows that overlapping peak is identified between anti-GR and anti-NCOA2 binding peaks in ChIP-seq-GSE99887 in the left panel. The middle Venn diagram shows that overlapping genes between overlapping genes of left Venn diagram and DEGs in mRNA-seq. The right Venn diagram shows that overlapping genes between the top 10% of Genecards-retrieval involved in macrophage polarization and overlapping genes of middle Venn diagram. The data are presented as mean ± SD.

In order to investigate the molecular basis in NCOA2-knock downed BMDMs treated with Ad-HNF4A, IL-4, and Dex, the mRNA-sequencing was performed. Principal component (PCA) analysis indicated that all samples were clustered in the shNC and shNCOA2 cells treated with Dex and IL-4, respectively. The difference between the shNC and shNCOA2 cells was statistically significant (Fig. 6B). The volcano plot showed that 1118 DEGs were upregulated and 1080 DEGs were downregulated under the screening standard of |Log2FC | >1 and Adjust *p* < 0.01 (Fig. 6C). As shown in the heatmap, it is demonstrated that the clustering relationships among the samples within each group are reliable. We found that some DEGs are related to the inflammatory response including Dpep1, Hmox1, Ccl25, Tusc2, Csf1, Stab1, and Pld3 (Fig. 6D). To better understand the role of these DEGs, we performed KEGG and GO enrichment analyses. GO results showed that DEGs are associated with regulation of inflammatory response, regulation of NF-kappaB transcription factor activity, and cell cycle DNA replication. KEGG results showed that DEGs were mainly related to the TNF, IL-17, and P13K-Akt signaling pathway (Fig. 6E). For the ChIP-seq database, 625 overlapping peaks were identified between anti-NCOA2 binding peaks and anti-GR binding peaks in GSE99887. The 625 overlapping binding peaks were identified by 533 genes. The analysis of Venn Diagram was conducted to detect the intersection of 533 genes and DEGs of mRNA-seq. 57 overlapping genes were obtained. Then, the analysis of Venn Diagram was performed to determine the intersection of 57 genes and top 10% of Genecards-retrieval “Macrophage polarization”. Finally, 10 genes were harvested (Fig. 6F). Of note, STAB1 has been reported to be associated with anti-inflammatory effects [25]. It has also reported that STAB1 knockdown enhances mortality and lung damage in CLP-induced septic mice [26]. Hence, we focused on exploring whether the HNF4A/NCOA2/GR axis influences macrophage polarization through STAB1 in a subsequent experiment.

### HNF4A/NCOA2/GR affects macrophage polarization through STAB1

**Fig. 7:**
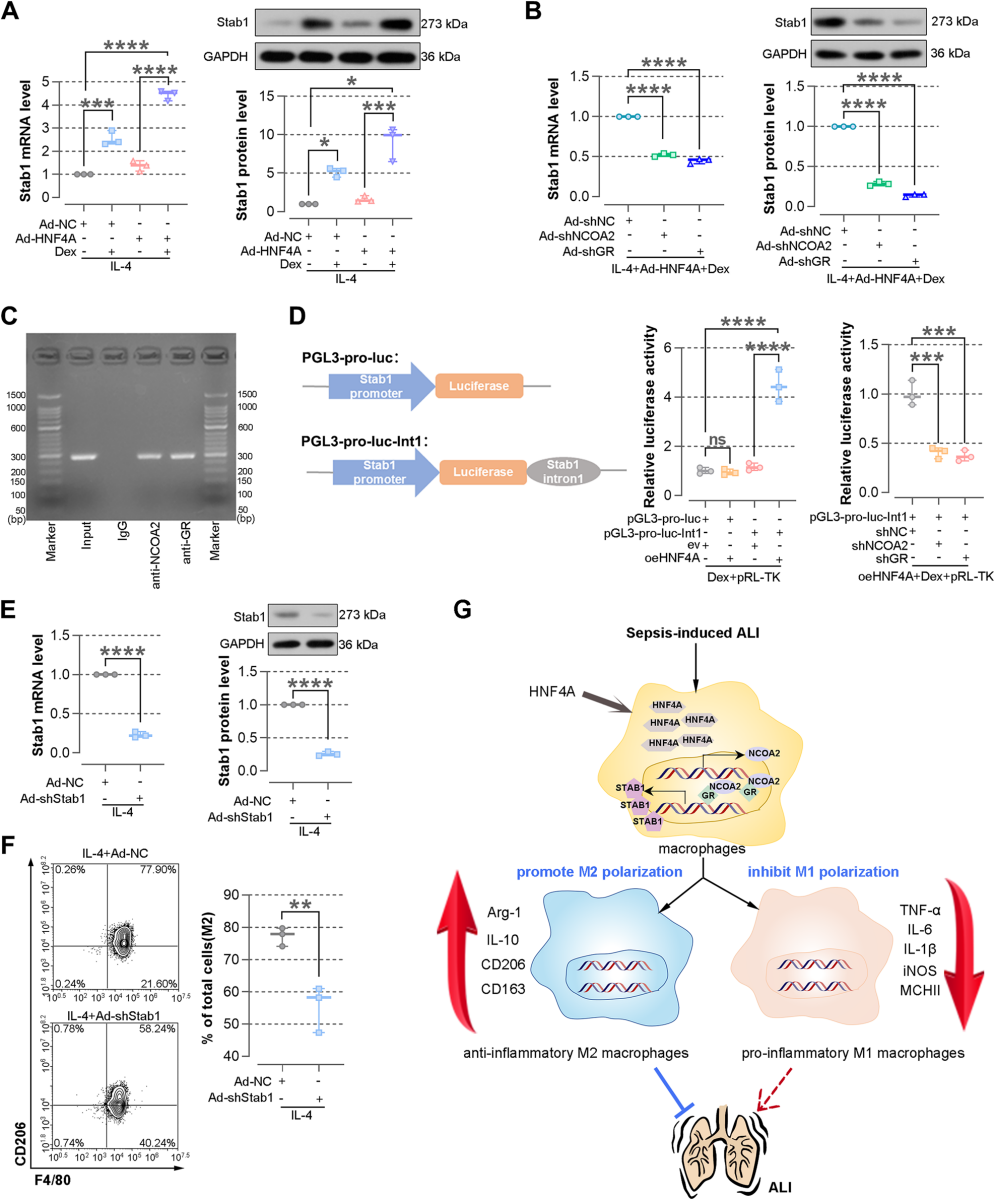
HNF4A/NCOA2/GR affects macrophage differentiation through STAB1. **A** BMDMs are infected with Ad-HNF4A for 48 h, and M2-type polarization of BMDMs is induced followed by the addition of 100 nM Dex. After 24 h, the expression of STAB1 in cells is detected by real-time PCR and WB. **B** BMDMs are infected with Ad-shNC, Ad-shNCOA2, or Ad-shGR for 48 h, and M2-type polarization of BMDMs is induced followed by the addition of 100 nM Dex. After 24 h, the expression of STAB1 in cells is detected by real-time PCR and WB. **C** M2-type polarization of BMDMs is induced followed by the addition of 100 nM Dex. ChIP assays to show that GR and NCOA2 bind to STAB1 intron 1. **D** pGL3-basic luciferase reporters containing STAB1 promoter and/or STAB1 intron1 are constructed. Dual luciferase reporter assays are performed. Luciferase activities are measured and normalized to the renilla activity. **E** After being infected with Ad-shNC or Ad-shSTAB1, M2-type polarization of BMDMs is induced. The expression of STAB1 is measured by real-time PCR and WB. **F** The expression of CD206 in BMDMs is measured by flow cytometry. **G** A possible mechanism for the treatment of sepsis is that HNF4A activates NCOA2 through transcription and promotes the transcriptional activation mediated by the NCOA2/GR complex, thereby promoting STAB1 expression. Schematics are drawn via SciDraw and Pinclipart. The lung and arrows are from Pinclipart; DNA: doi.org/10.5281/zenodo.3926245. The data are presented as mean ± SD. *P < 0.05, **P < 0.01, ***P < 0.001, ****P < 0.0001.

To investigate whether STAB1 is involved in the effect of HNF4A/NCOA2/GR on macrophage polarization, BMDMs were infected with Ad-shSTAB1 to knockdown STAB1 in BMDMs treated with IL-4. This was confirmed by real-time PCR, WB (Fig. 7E). The results of flow cytometry suggested that knockdown of STAB1 inhibits macrophage polarization towards M2 phenotype (Fig. 7F). Conversely, knockdown of STAB1 increased the level of TNF-α in BMDMs treated with LPS and IFN-γ, indicating that knockdown of STAB1 promotes macrophage polarization towards M1 phenotype (Supplementary Fig. 2L). In the activated state of GR pathway, STAB1 levels were markedly upregulated after HNF4A overexpression (Fig. 7A). Under the conditions of adding Dex and overexpressing HNF4A, knocking down NCOA2 or GR downregulated the levels of STAB1, indicating that STAB1 may a target of the HNF4A/NCOA2/GR axis (Fig. 7B). Interestingly, ChIP-PCR and dual luciferase assays validated that GR/NCOA2 bind to STAB1 intron 1 (Fig. 7C, D). The above results show that HNF4A/NCOA2/GR affects macrophage polarization through STAB1. Overall, this study revealed that the regulatory axis of HNF4A/NCOA2/GR/STAB1 affects macrophage polarization, thereby improving sepsis-induced lung damage (Fig. 7G).

## Discussion

Sepsis-induced ALI is a serious clinical complication that can be life-threatening in severe cases [27]. Currently, the treatment management of ALI in sepsis is progressing towards a more precise and integrated mode, involving advanced methods such as antioxidant therapy, management of cell death, carbon monoxide delivery strategies, and interventions focused on specific disease processes [28, 29]. In-depth investigations at the molecular level are crucial for identifying novel therapeutic targets and prediction tools for the lung injury in sepsis. In this study, we identified a new molecule, HNF4A, as a regulator affecting lung injury in sepsis.

By analyzing a CLP-induced microarray of gene expression in lung tissue of sepsis mice, we found that a notable reduction in the expression of HNF4A. HNF4A is a transcription factor necessary for the development of the liver, playing a significant role in maintaining the benign phenotype [30, 31]. Recent research has also suggested its involvement in inflammation [18] and macrophage polarization [16]. In this study, we observed diminished HNF4A expression in the lung tissues in a murine model of sepsis. Remarkably, our findings also revealed that HNF4A was expressed in alveolar macrophages, and its expression was consistently decreased in macrophages obtained from alveolar lavage fluid of the CLP mice as compared to normal mice. To further verify the role of HNF4A, we treated the septic mice with an adenovirus-mediated HNF4A overexpression intervention. The administration of HNF4A-overexpressed adenoviral particles in mice raised the expression of HNF4A in the lung and reduced the sepsis-induced mortality. Moreover, HNF4A overexpression mitigates the CLP-induced pulmonary edema and lung barrier damage and reduces inflammatory cell infiltration, such as neutrophils and macrophages.

Macrophages in the lung, involving alveolar macrophages and interstitial macrophages, are immune cells that reside in lung tissues and play a crucial role in immune defense and maintaining the tissue homeostasis [32]. Through their high plasticity, these macrophages can display pro-inflammatory or anti-inflammatory characteristics depending on the different microenvironmental stimulation, which is vital for the lung health [33]. Proinflammatory macrophages, also known as M1-phenotype macrophages, are an activated state of macrophages. In this state, macrophages display strong pro-inflammatory activities and produce pro-inflammatory cytokines (such as TNF-α, IL-1β, and IL-6) as well as express inducible nitric oxide synthase (iNOS) to eradicate pathogens and promote inflammation. Anti-inflammatory macrophages, commonly referred to as M2-phenotype macrophages, are another important activation state of macrophages. Macrophages in this state release anti-inflammatory cytokines (such as IL-10) to participate in tissue repair, immune regulation, anti-inflammatory response, and angiogenesis [34, 35]. They are also involved in the maintenance of tissue homeostasis and immune tolerance by expressing specific surface markers such as CD206 and CD163 [36]. In this work, we demonstrated that HNF4A overexpression facilitated the transition of macrophages from the M1 phenotype triggered by CLP to the M2 phenotype. This transition was accompanied by alterations in the expression of pro-inflammatory cytokines TNF-α, IL-1β, IL-6, M1 surface markers MHC II, anti-inflammatory cytokines IL-4 and IL-10, and M2 surface markers CD206, CD163, and Arg-1.

In terms of downstream regulators of HNF4A, our attention was directed on NCOA2. As a transcription coactivator, this protein has been demonstrated to regulate septic injury [37] and macrophage polarization [24]. In this study, we noted the existence of NCOA2 in lung macrophages, and the level of NCOA2 expression was also elevated in lung tissues and macrophages after HNF4A overexpression. Considering the characteristics of HNF4A as a transcription factor, we employed the dual luciferase assay and ChIP-PCR to confirm the interaction between the NCOA2 promoter and HNF4A. The experiments demonstrated that HNF4A enhances the expression of NCOA2 by promoting its transcription. Additionally, as a transcriptional coregulator, the NCOA2 has been defined to bind with GR [23] to regulate gene expression. Notably, the downregulation of NCOA2 in macrophages counteracts the transcriptional suppression of pro-inflammatory proteins mediated by GR [23]. Previous studies have also shown that conditional knockout of NCOA2 [37] or GR [38] leads to an increased mortality rate in mice suffering sepsis. These studies suggest that the NCOA2-GR pathway may be a target of the HNF4A impact. To verify this hypothesis, we utilized a glucocorticoid receptor (GR) ligand, Dex, as an activator of the GR pathway for the investigation. Dex greatly increased the ability of HNF4A to induce macrophages transferring into the M2 phenotype and decreased the ability of HNF4A to induce macrophages transferring into the M1 phenotype. Nevertheless, when NCOA2 is suppressed, the impact of HNF4A is largely canceled, while Dex could restore the promotion of HNF4A on the M2 polarization of macrophages and the inhibition of HNF4A on the M1 polarization of macrophages. These phenomena suggest that HNF4A has a regulatory function in the process of macrophage M2 and M1 polarization through the NCOA2-GR pathway.

In addition, we employed transcriptome sequencing to investigate the genes influenced by HNF4A/NCOA2/GR axis. More than 2000 genes exhibiting differential expression were identified, and following a series of screenings, our attention was directed towards STAB1. STAB1, also known as macrophage-stimulating 1 receptor, is a transmembrane protein expressed on a variety of cell types, especially macrophages [39]. It is involved in regulating immune response and inflammatory processes, as well as tissue repair and metabolic balance [40]. By recognizing different ligands such as collagen fragments and heparan sulfate [41], STAB1 can affect the polarization of macrophages and promote the transformation to M2 phenotype, thus playing a crucial role in inflammation suppression and tissue healing [42]. In the current study, we observed that HNF4A promoted the expression of STAB1, while NCOA2 or GR silencing inhibited the STAB1 expression raised by HNF4A. Moreover, STAB1 knockdown promoted the M1 phenotypic switch of macrophages and suppressed the M2 phenotypic switch of macrophages. Unexpectedly, the mechanism by which NCOA2/GR regulates STAB1 is not directly linked to the promoter of STAB1. This is evident from the fact that overexpression of HNF4A does not significantly affect the luciferase activity of PGL3-pro-luc, which represents the promoter activity of STAB1. However, the NCOA2/GR complex may bind to the first intron of STAB1, which contains the GR element. The luciferase results confirmed that HNF4A increased the promoter activity containing the first intron of STAB1.

In conclusion, this research validates the potential protective effect of HNF4A against lung injury in sepsis. HNF4A may transcriptionally activate the NCOA2 promoter and increase its expression, thus, in turn, increasing the formation of NCOA2/GR complex and inducing gene transcription such as STAB1 to promote the polarization of macrophages towards M2 and reduce the polarization of M1. As a result, the production of inflammatory factors and the infiltration of inflammatory cells are inhibited, leading to the alleviation of lung injury induced by sepsis. The regulatory axis of HNF4A/NCOA2/GR/STAB1 could potentially serve as a therapeutic target for sepsis-associated lung damage.

## Materials and methods

### Induction of sepsis-associated lung injury in mice

Before modeling, female and male C57BL/6 J mice aged 8–12 weeks were adaptively fed for two weeks. The mice had free access to food and water. Mice were maintained under temperature 20–26 °C, relative humidity 40–60%. The sepsis model was established by CLP. Briefly, C57BL/6 J mice (half male and half female) were randomly allocated to the Sham and CLP groups (*n* = 6/group). Mice were anesthetized via inhalation isoflurane. The cecum was ligated and then punctured twice with a 25G-gauge syringe needle in mice of CLP group. After that, the cecum was placed back in the original position, and the incision was closed. The similar surgery without CLP was performed on mice of the sham group. Besides survival analyses, the mice were subjected to euthanasia 8 h after operation, and lung tissues along with alveolar lavage fluid were harvested. The concentration of total protein in the alveolar lavage fluid was quantified utilizing a BCA kit (Beyotime Biotech, Shanghai, China). A portion of the lung tissues was used to assess wet and dry weights, from which the ratio of wet/dry weight was calculated. Additional lung tissue samples were fixed with neutral formaldehyde solution or frozen with liquid nitrogen for subsequent experiments. For survival assays, the survival status of mice was observed every 12 h for 10 days. Accidental deaths during the experiment were excluded from the study. Sample sizes were designed with power of 80% at an alpha of 0.05 according to the literature and our previous studies. In this study, blinding was implemented to minimize bias and ensure the objectivity of the results in histological pathology experiments and molecular detection experiments.

### Hematoxylin and eosin (H&E) staining and scoring

The dehydrated lung tissues were embedded in paraffin and cut into 5 μm-thick sections. These sections were dewaxed in dimethylbenzene and dehydrated in a concentration gradient of alcohol. Subsequently, lung slides were stained with H&E. Lung slides were dehydrated, permeabilized, and sealed prior to being visualized using a microscope (BX53; Olympus, Tokyo, Japan) under 200× magnifications. The criteria utilized for assessing tissue damage are based on methods established in prior research [43]. The infiltration of inflammatory cells was observed and the injury of lung tissues was scored.

### Real-time PCR

Total RNA was extracted from lung tissues, or cells using the TRIpure reagent (BioTeke Corporation, Beijing, China). The concentration of RNA in each sample was determined using ultraviolet spectrophotometer (NANO 2000, Thermo Scientific, Pittsburgh, PA, USA). RNA samples were reversely transcribed to obtain the corresponding cDNA. After that, the fluorescence quantitative PCR instrument (Bioneer, Daejeon, Korea) was used to perform the real-time PCR assay. The final relative expression of mRNA was determined utilizing the 2^−△△CT^ method. The primers are provided in Table 1.

**Table 1:** primer sequences used in real-time PCR.

primer	Forward	Reverse
mus Arg-1 mus CD163 mus CD206 mus HNF4A mus NCOA2	TATCTGCCAAAGACATCG CAGCTTCGCTTGGTAGG GCAAGTGATTTGGAGGCT TGGTCGAGTGGGCCAAGTA CTCATCCGTTCTCAGACTACTA	ATCACCTTGCCAATCCC CACATTGGCATCAGTCATA ATAGGAAACGGGAGAACC GTGCCGAGGGACGATGTAG ATGCCCATTTGTTCCTTT
mus Stab1 mus iNOS mus MHCII mus GAPDH	TGCTTCACTCCCTCAACT CACCACCCTCCTCGTTC CAGCCAAGATCAAAGTGCG TGTTCCTACCCCCAATGTGTCCGTC	CCCAAGGCTAACTACACC CAATCCACAACTCGCTCC GCCAAGCCCGAGGAAGA CTGGTCCTCAGTGTAGCCCAAGATG

### Western blot analysis

Total proteins in tissues or cells were extracted and quantified using a BCA kit (Solarbio, Beijing, China). The proteins were subjected to separation via SDS-PAGE, followed by transferring onto PVDF membranes. After blocking, the membranes were incubated with the primary antibodies at 4 °C overnight, and then with the secondary antibodies at 37 °C for 1 h. After visualization with the ECL reagent (Solarbio, Beijing, China), the optical density values of the protein bands were analyzed using the Gel-Pro-Analyzer software. The primary antibodies were as follows: HNF4A antibody (1:500, bs-3828R, Bioss, Beijing, China), NCOA2 antibody (1:1000, bs-20558R, Bioss, Beijing, China) and STAB1 antibody (1:500, bs-7510R, Bioss, Beijing, China). The secondary antibodies were as follows: HRP-labeled goat anti-rabbit (1:3000, SE134, Solarbio, Beijing, China) IgG and HRP-labeled goat anti-mouse IgG (1:3000, SE131, Solarbio, Beijing, China).

### Immunofluorescence (IF) staining

Lung tissues were made into 5 μm-thick paraffin sections. After dewaxing, antigen repairing, and blocking, the lung sections were incubated overnight with diluted F4/80 antibody (1:50, Sc-377009, Santa Cruz, Dallas, TX, USA), NCOA2 antibody (1:100, bs-20558R, Bioss, Beijing, China), or HNF4A antibody (1:100, bs-3828R, Bioss, Beijing, China) overnight at 4 °C. For samples of alveolar macrophages, cell sections were fixed for 15 min and incubated with 0.1% tritonX-100 (Beyotime Biotech, Shanghai, China) for 30 min at room temperature, followed by incubation with NCOA2 antibody (1:100, bs-20558R, Bioss, Beijing, China). Then sections were incubated with Cy3-labeled goat anti-rabbit IgG (1:200, ab6939, Abcam, Cambridge, UK) and FITC-labeled goat anti-mouse IgG (1:200, ab6785, Abcam, Cambridge, UK) for 90 min at room temperature. DAPI was used to counterstain the nuclei. After dropping the anti-fluorescence quenching sealing reagent, the sections were photographed under a fluorescence microscope (BX53; Olympus, Tokyo, Japan) under 400× magnifications.

### Construction of adenovirus vectors

Adenoviruses overexpressing HNF4A (Ad-HNF4A) or negative controls (Ad-NC) were generated. For mouse experiments, Ad-NC or Ad-HNF4A of 1 × 10^9^ pfu was instilled into the trachea of each mouse 48 h prior to establishing the model. For cell experiments, cells were cultured in the medium containing Ad-NC or Ad-HNF4A for 48 h. Bone marrow-derived macrophages (BMDMs) were treated with IL-4 for 24 h to induce M2 polarization. The 100 nM dexamethasone (Dex) was added to activate the GR pathway. shNCOA2 and shGR were synthesized to knock down the expression of NCOA2 and GR by adenovirus delivery to infected macrophages.

### Enzyme-linked immunosorbent assay (ELISA)

To investigate the effect of HNF4A on the inflammation of lung tissue in septic mice, ELISA was performed on alveolar lavage fluid to detect IL-1β, IL-6, TNF-α, IL-4, and IL-10 via the corresponding kits (Multi Sciences, Hangzhou, China). OD values at 450 nm and 570 nm were determined and the corresponding concentrations were calculated.

### Wright-Giemsa staining

Alveolar macrophages were isolated from alveolar lavage fluid. After washing with PBS, the alveolar lavage solution was centrifuged at 300 × *g* for 5 min. Subsequently, cells were seeded in a medium containing 10% fetal bovine serum. Alveolar macrophages were stained with a Wright-Giemsa stain kit (Jiancheng Bioengineering Institute, Nanjing, China). In brief, alveolar lavage fluid preserved by centrifugation was resuspended. Cell slides were made and fixed in methanol for 15 min. After drying, the slides were stained with Giemsa A for 1 min and Giemsa B for 7 min. Ultimately, the cells were destained with 80% ethanol until the cells were clear. Total cells, neutrophils, and macrophages in alveolar lavage fluid were counted respectively.

### Flow cytometer analysis

To detect M1-type or M2-type polarization, macrophages in alveolar lavage fluid of mice were incubated with F4/80 antibody (F21480A03, Multi Sciences, Hangzhou, China), Arg-1 antibody (12-3697-80, Thermo Scientific, Pittsburgh, PA, USA) or TNF-α antibody (12-27321-41, Thermo Scientific, Pittsburgh, PA, USA) at 4 °C for 30 min in the darkness. BMDMs were incubated with F4/80 antibody and CD11b antibody (F41011b02, Multi Sciences, Hangzhou, China) to identify the macrophages. BMDMs were incubated with F4/80 antibody and CD206 antibody (12-2061-80, Invitrogen, Carlsbad, CA, USA) to measure M2-type polarization. Subsequently, flow dye buffer was added and the sample was centrifuged (300 g) for 5 min. The supernatant was removed and resuspended with flow dyeing buffer assaying by flow cytometry.

### Isolation and culture of mouse BMDMs

BMDMs were obtained using the previous method [44]. In the cell experiment, three biological replicates were performed. The mouse femur was removed in a sterile state and cleaned with PBS. The two ends of the bone were cut with sterile scissors, and the bone marrow was rinsed from one end with a syringe needle containing serum-free medium so that the liquid flowed out from the other end and was received into a sterile centrifuge tube for 10 min. After the cell precipitation was suspended with the supernatant of L929 cells, it was inoculated in a culture dish and cultured in an incubator at 37 °C and 5% CO_2_. L929 cells were purchased from iCell and cultured in minimum essential medium containing 10% equine serum. The cells were identified by STR and tested negative for mycoplasma contamination.

### Dual-luciferase reporter assay

The NCOA2 promoter fragment was inserted into the pGL3-basic luciferase vector to generate the pGL3-NCOA2 promoter luciferase reporter. 293 T cells were co-transfected with pGL3-NCOA2 promoter luciferase reporter and pRL-TK renilla luciferase reporter plasmid. STAB1 promoter was inserted into the upstream of the transcription unit in the pGL3-basic vector to generate the PGL3-pro-luc promoter luciferase reporter. STAB1 promoter is inserted into the upstream of the transcription unit in the pGL3-basic vector, and STAB1 intron1 is inserted into the downstream of the transcription unit in the same vector to generate the PGL3-pro-luc-Int1 promoter luciferase reporter. The cells were lysed 48 h after transfection. Firefly luciferase and renilla luciferase activities were determined by the dual luciferase reporter gene assay kit (KeyGEN, Nanjing, China). The relative luciferase activity was normalized with renilla luciferase activity. 293 T cells were cultured with DMEM containing 10% FBS in a 37 °C, 5% CO_2_ incubator. The cells were purchased from iCell.

### Chromatin immunoprecipitation (ChIP) assay

The binding of HNF4A to NCOA2 promoter was detected using the ChIP assay kit (Beyotime, Shanghai, China). Briefly, DNA and protein were cross-linked using 1% formaldehyde. The Cells were lysed and sonicated to shear DNA. Cell lysates were immunoprecipitated with control rabbit IgG or HNF4A antibody (Bioss, Beijing, China). PCR was used to amplify the purified DNA from the cell lysates and the DNA recovered from the immunoprecipitation. The primers for NCOA2 are 5′-GGGAGGCACCTCTGGGACTA-3′ (forward) and 5′-CCTGGCTGGCGAGACTTCA-3′ (reverse); STAB1 intron1: 5′-TCCTTGGCTGACAGTGGG-3′ (forward) and 5′-CTGGTGGGCAGATGAGTG-3′ (reverse).

### mRNA-seq

BMDMs were transfected with Ad-HNF4A and/or Ad-shNCOA2 for 48 h, followed by addition of IL-4 and Dex. Total RNA was isolated from cell samples, after which complementary DNA (cDNA) libraries were constructed and subjected to mRNA sequencing (mRNA-seq). DEGs were defined as |log2FoldChange | >1 and Adjust *p* < 0.01. Subsequently, all DEGs were subjected to analysis for Gene Ontology (GO) and Kyoto Encyclopedia of Genes and Genomes (KEGG) enrichment, with the aim of conducting functional annotation.

### Statistical analysis

Data were presented in the form of mean ± standard error of mean. GraphPad Prism version 9.5 was utilized for the purposes of data visualization and statistical analysis. The data were tested for normality and homogeneity of variance. The differences between two groups were evaluated using two-tailed unpaired Student *t*-tests. The differences among three or more groups were evaluated using One-way ANOVA post hoc Tukey’s tests. The survival curve was compared by the log-rank test and plotted by the Kaplan–Meier method. All analysis with *p* < 0.05 were considered significant.

## Supplementary information

Supplemental Material

Original Data

Original real-time PCR

## Data Availability

Data deposition The mRNA-seq data was presented in Dryad database (10.5061/dryad.gf1vhhn06). Data will be made available on reasonable request from the corresponding author.
