# Correction: HNF4A mitigates sepsis-associated lung injury by upregulating NCOA2/GR/STAB1 axis and promoting macrophage polarization towards M2 phenotype

**DOI:** 10.1038/s41419-025-07519-x

**Published:** 2025-04-14

**Authors:** Yu-Hang Yang, Ri Wen, Xin-Mei Huang, Tao Zhang, Ni Yang, Chun-Feng Liu, Tie-Ning Zhang

**Affiliations:** 1 Department of Pediatrics, PICU, Shengjing Hospital of China Medical University, Shenyang, China; 2 Department of Endocrinology, Shanghai Fifth People’s Hospital, Fudan University, Shanghai, China

**Keywords:** Bacterial infection, Inflammatory diseases

Correction to: *Cell Death and Disease* (2025)16:120 10.1038/s41419-025-07452-z, published online 21 February 2025

In this article here are three instances in this article where “NCOR2” should be corrected to “NCOA2.” The corresponding sentences should be revised as follows:

1. Title: “HNF4A mitigates sepsis-associated lung injury by upregulating NCOA2/GR/STAB1 axis and promoting macrophage polarization towards M2 phenotype”
2. The last sentence of the abstract: “It is suggested that one of the regulatory mechanisms involved in this association may be the NCOA2/GR/STAB1 axis.”
3. Discussion in paragraph 5, following reference [42]: “In the current study, we observed that HNF4A promoted the expression of STAB1, while NCOA2 or GR silencing inhibited the STAB1 expression raised by HNF4A.”

The original article has been corrected.

