# Supplementary material for: HNF4A mitigates sepsis-associated lung injury by upregulating NCOA2/GR/STAB1 axis and promoting macrophage polarization towards M2 phenotype
Source: Cell Death Dis. 2025 Feb 21;16(1):120. doi: 10.1038/s41419-025-07452-z (PMC11842871; doi:10.1038/s41419-025-07452-z)
Supplement: Supplementary file 1 — Supplemental Material [file 41419_2025_7452_MOESM1_ESM.docx]

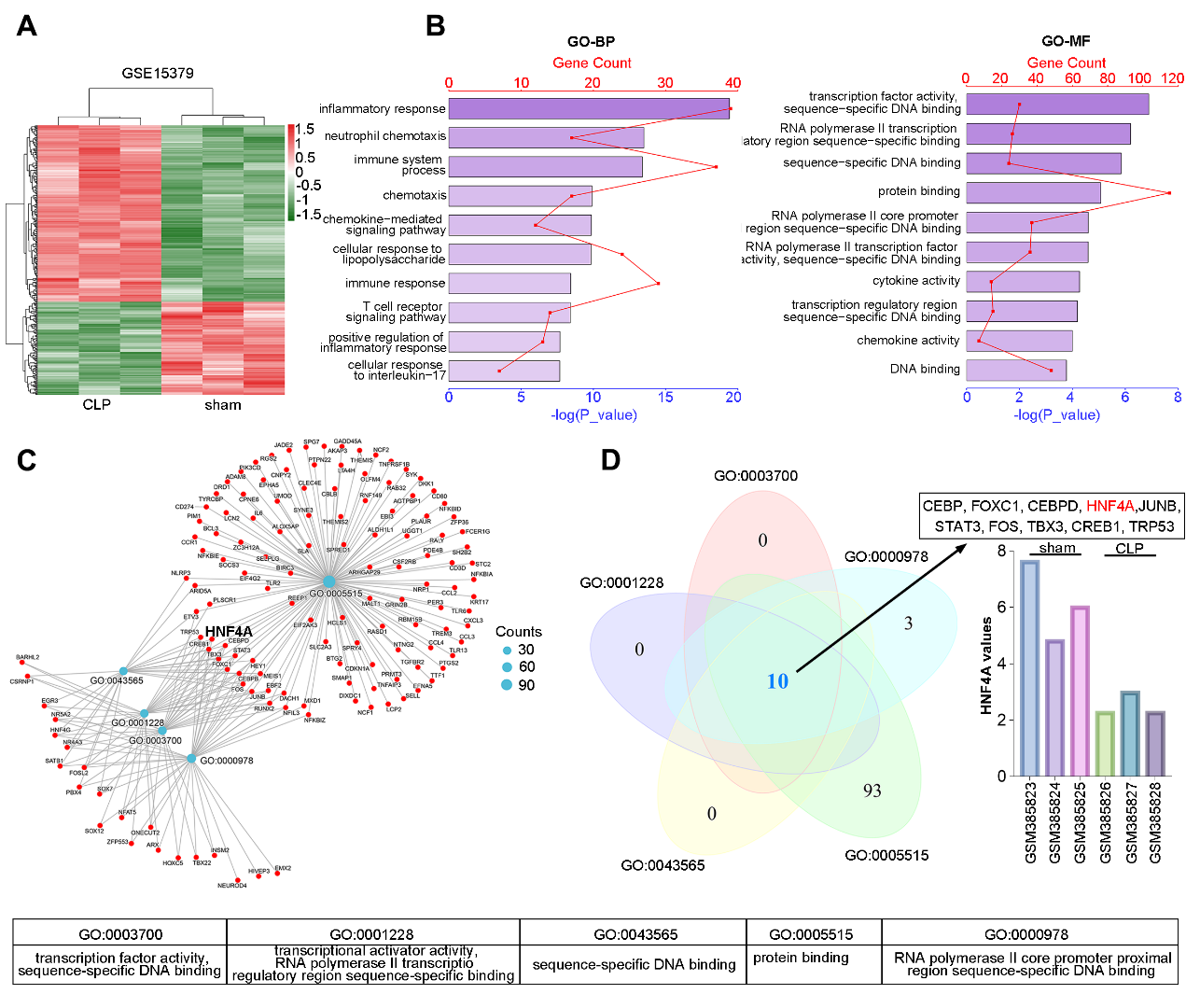


**Supplementary Figure1. Transcriptomic analysis of lung tissue from CLP-induced septic mice and sham mice in GSE15379**

(A) Heatmap of DEGs in CLP-induced septic mice and sham mice identified in GSE 15379. (B) Top 10 of GO enrichment terms in BP and MF. (C) The enrichment results of DEGs are constructed with enrichment network which shows the top 5 of GO enrichment terms on MF levels. (D) Venn diagram shows overlapping genes counts in the top 5 of GO enrichment terms on MF levels. Right panel shows HNF4A expression values in CLP-induced samples and sham samples in GSE15379.


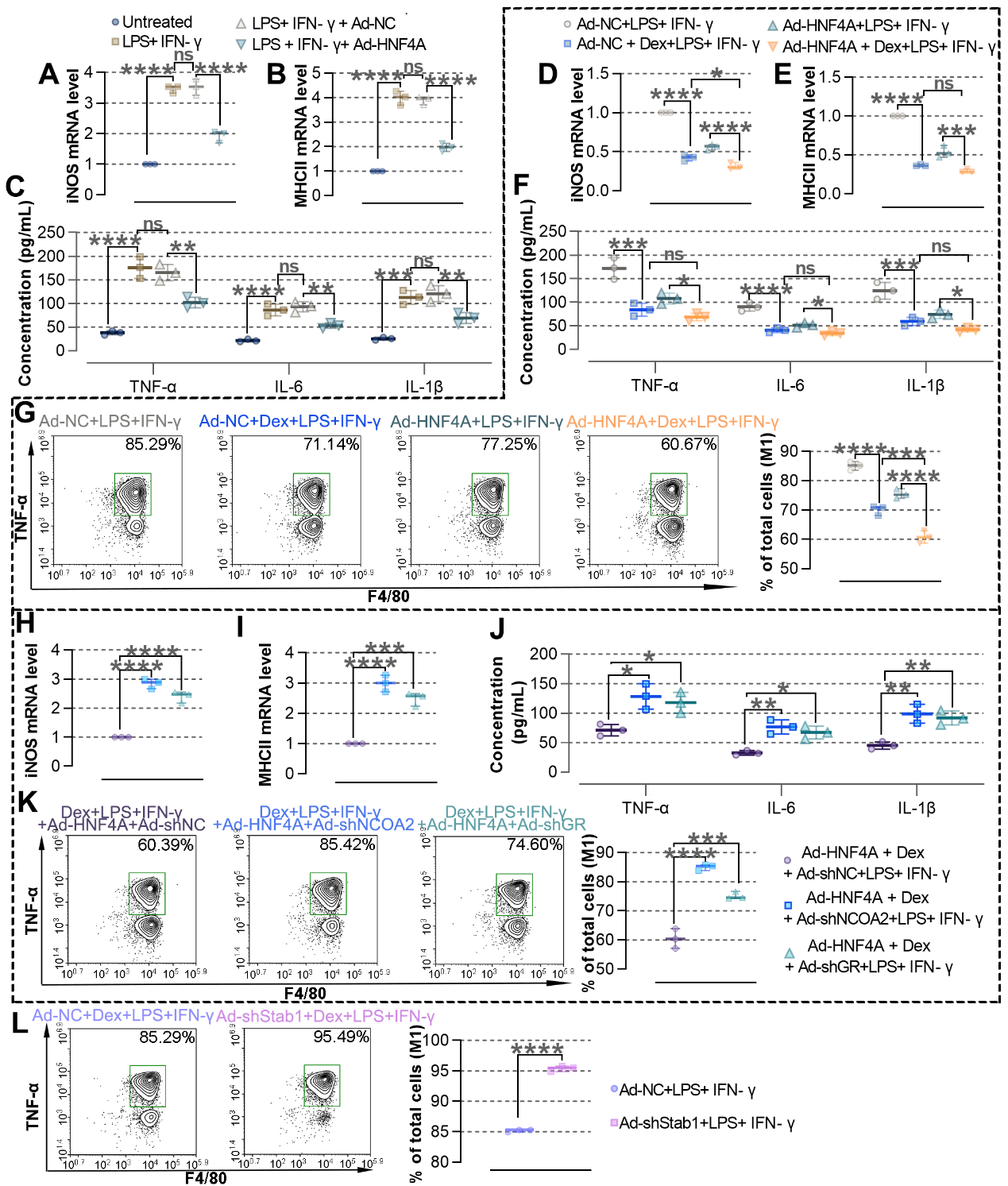


**Supplementary Figure2. Analysis of M1-type polarization in BMDMs.**

BMDMs are infected with Ad-NC or Ad-HNF4A for 48 h, and then M1-type polarization of BMDMs is induced with LPS and IFN-γ. After 24 h, the levels of iNOS (A) and MHCII (B) in cells are detected by Real-time PCR. (C) The levels of inflammatory factors (TNF-α, IL-1β, and IL-6) are detected in BMDMs. BMDMs are infected with Ad-NC or Ad-HNF4A for 48 h, and then cells are cultured with LPS, IFN-γ and Dex for 24 h. The levels of iNOS (D) and MHCII (E) in cells are detected by Real-time PCR. (F) The levels of inflammatory factors (TNF-α, IL-1β, and IL-6) are detected in BMDMs. (G) The expression of TNF-α in BMDMs is detected by flow cytometry. BMDMs infected with Ad-HNF4A are infected with Ad-shNCOA2, Ad-shNC and Ad-shGR for 48 h, and then cells are cultured with LPS, IFN-γ and Dex for 24 h. The levels of iNOS (H) and MHCII (I) in cells are detected by Real-time PCR. (J) The levels of inflammatory factors (TNF-α, IL-1β, and IL-6) are detected in BMDMs. (K) The expression of TNF-α in BMDMs is detected by flow cytometry. BMDMs are infected with Ad-NC or Ad-shStab1, and then cells are cultured with LPS, IFN-γ and Dex for 24 h. (L) The expression of TNF-α in BMDMs is detected by flow cytometry. The data are presented as mean ± SD. **P* < 0.05, ***P* < 0.01, ****P* < 0.001, *****P* < 0.0001.
