# Supplementary material for: HNF4A mitigates sepsis-associated lung injury by upregulating NCOA2/GR/STAB1 axis and promoting macrophage polarization towards M2 phenotype
Source: Cell Death Dis. 2025 Feb 21;16(1):120. doi: 10.1038/s41419-025-07452-z (PMC11842871; doi:10.1038/s41419-025-07452-z)
Supplement: Supplementary file 2 — Original Data [file 41419_2025_7452_MOESM2_ESM.pdf]

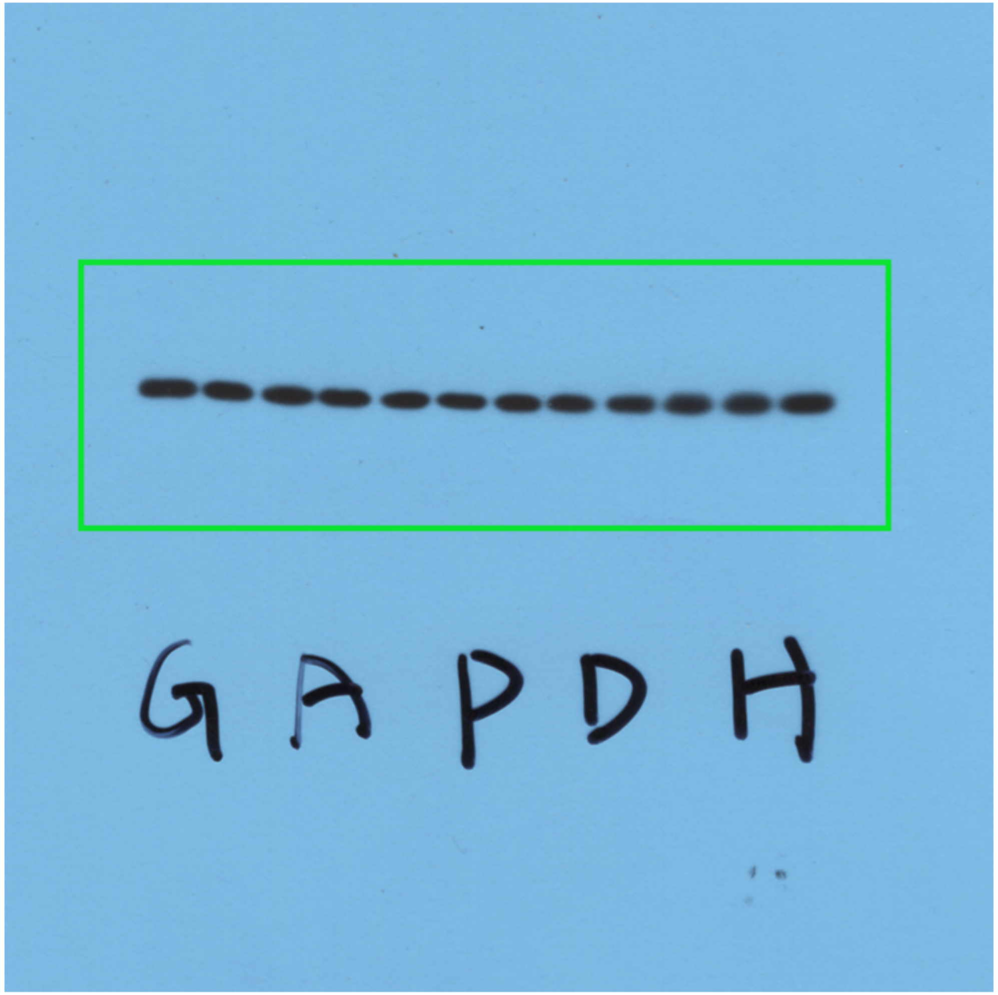
GAPDH

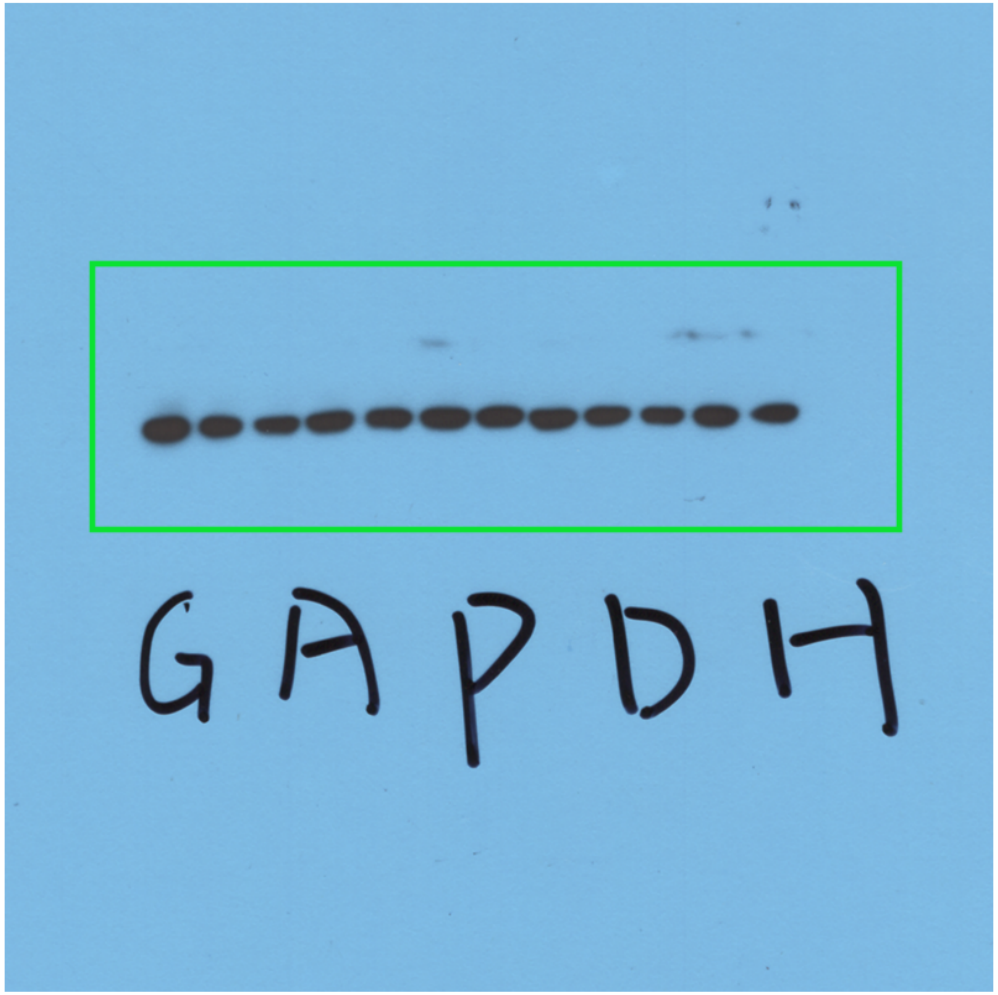
GAPDH

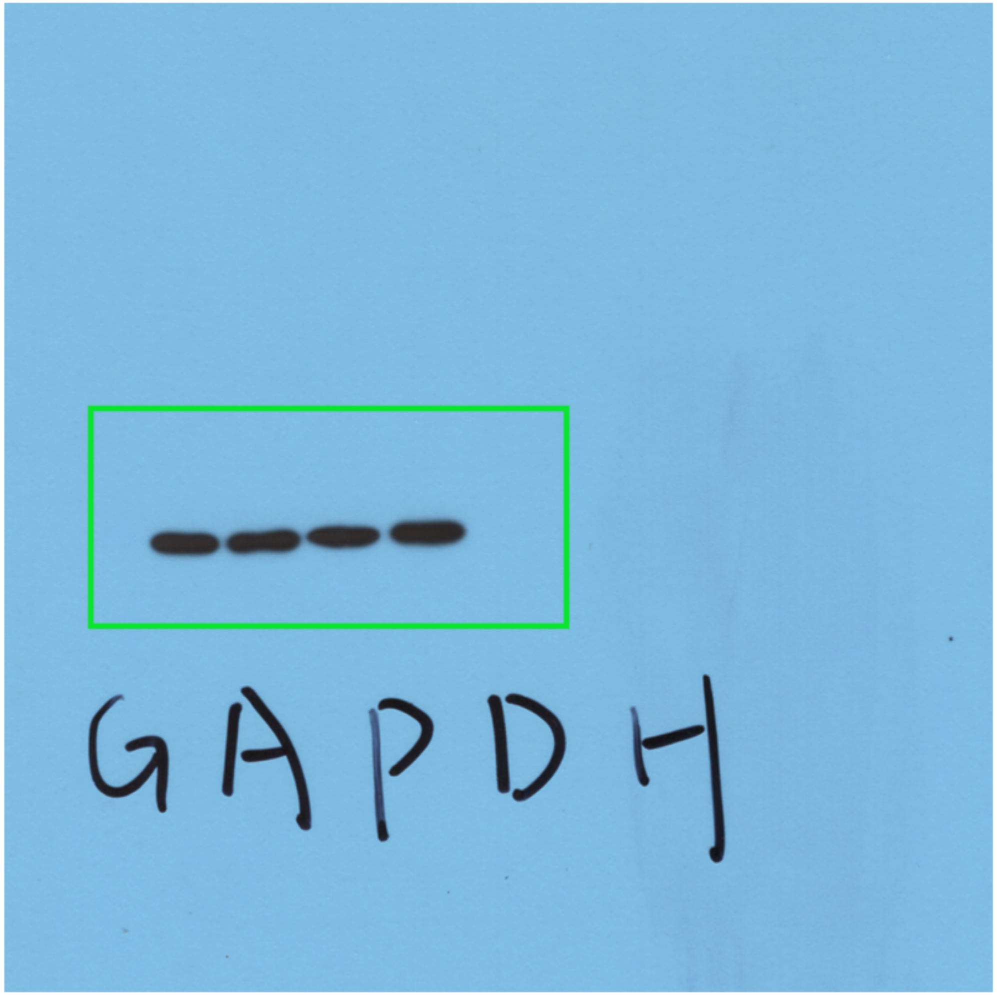
GAPDH

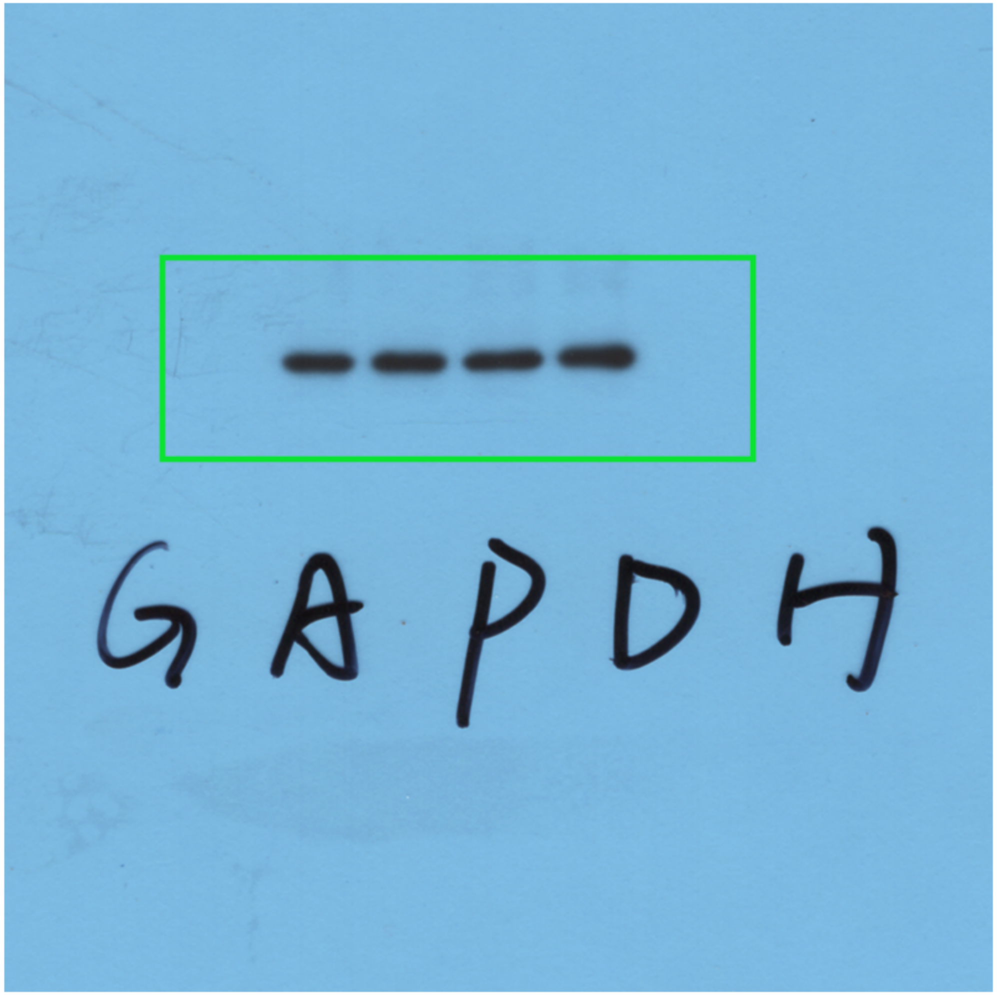
GAPDH

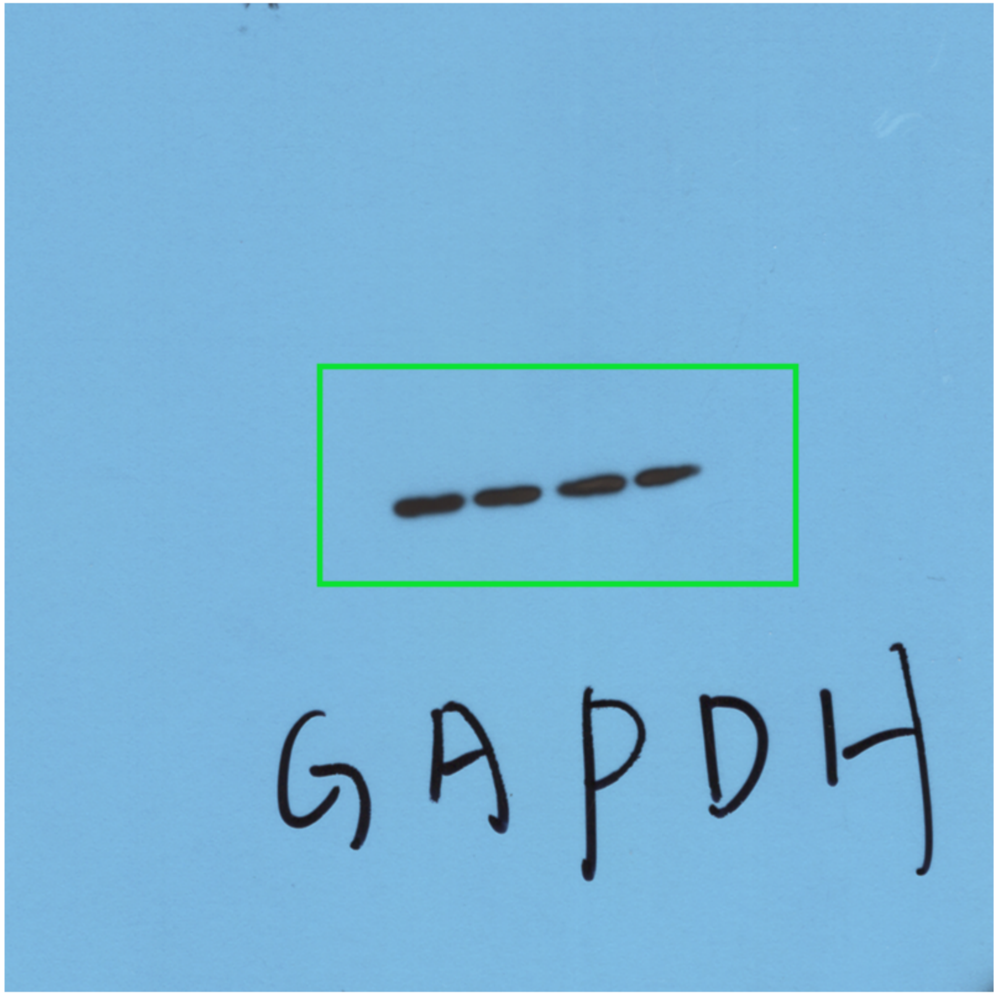
GAPDH

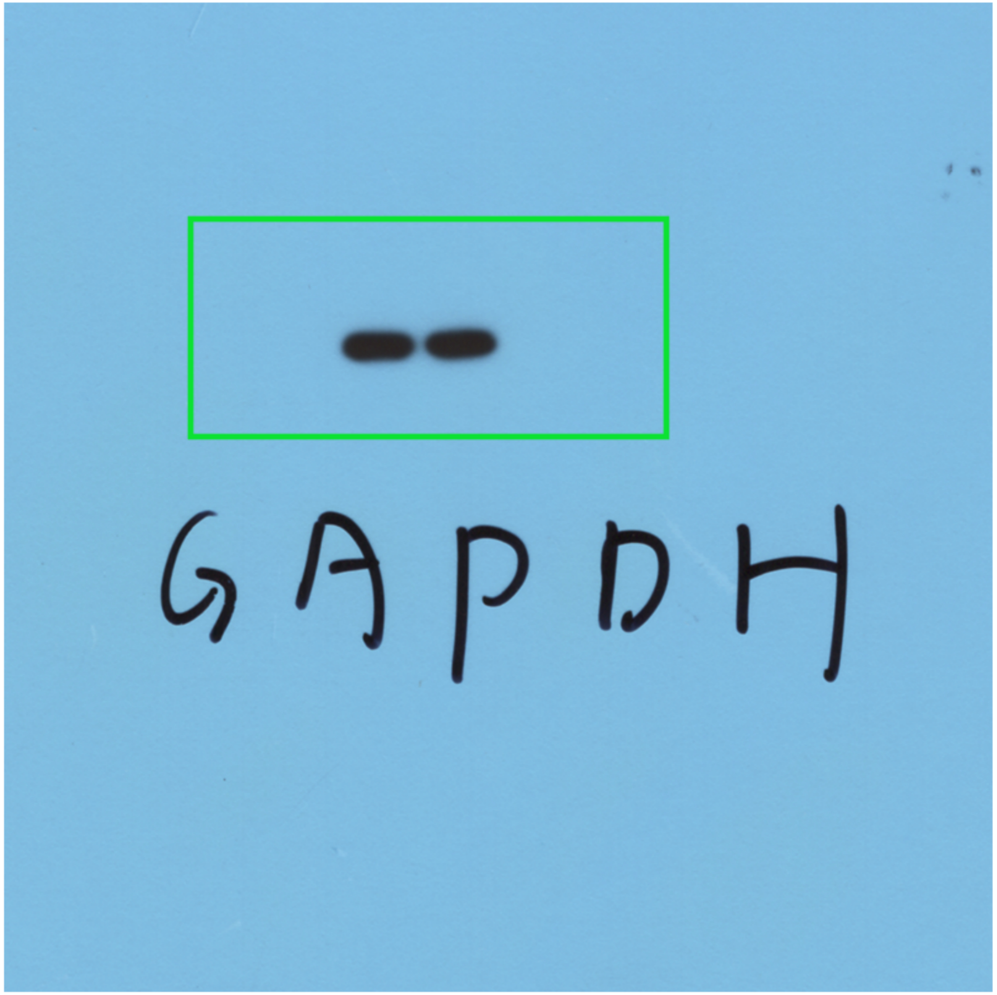
GAPDH

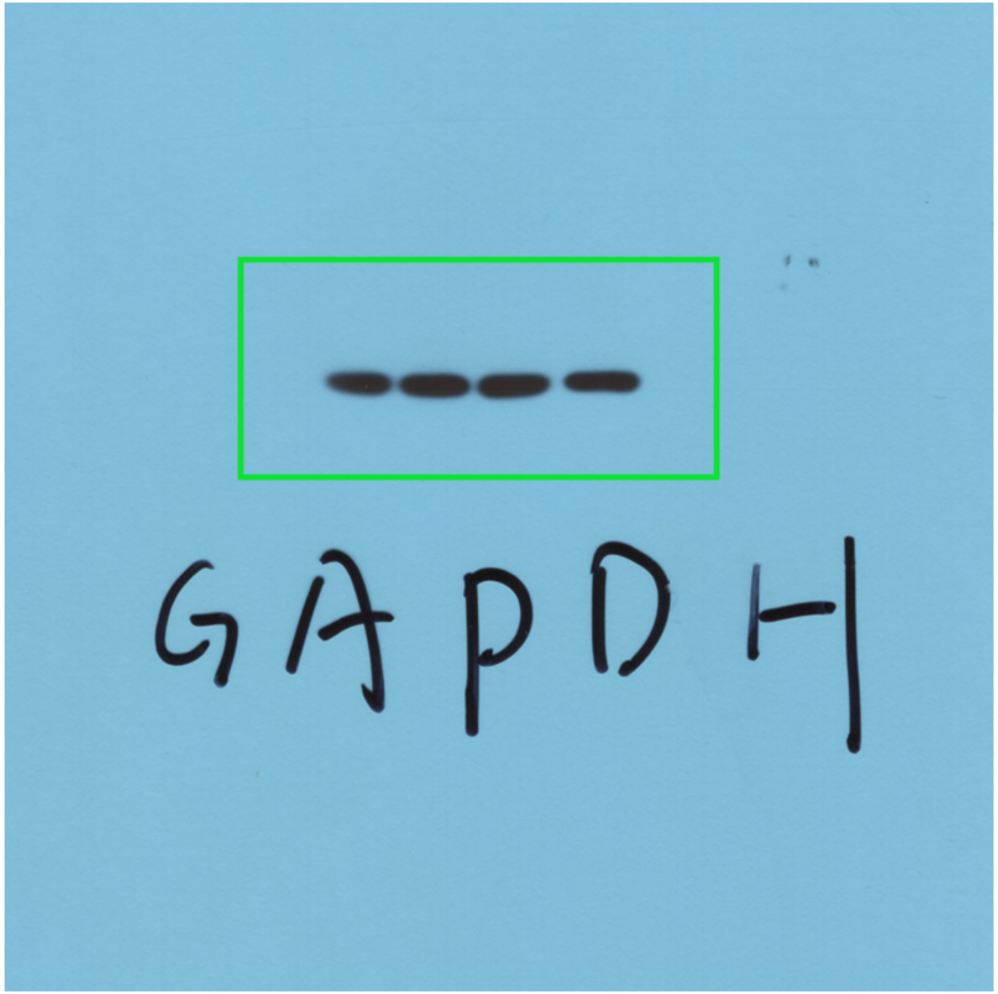
GAPDH

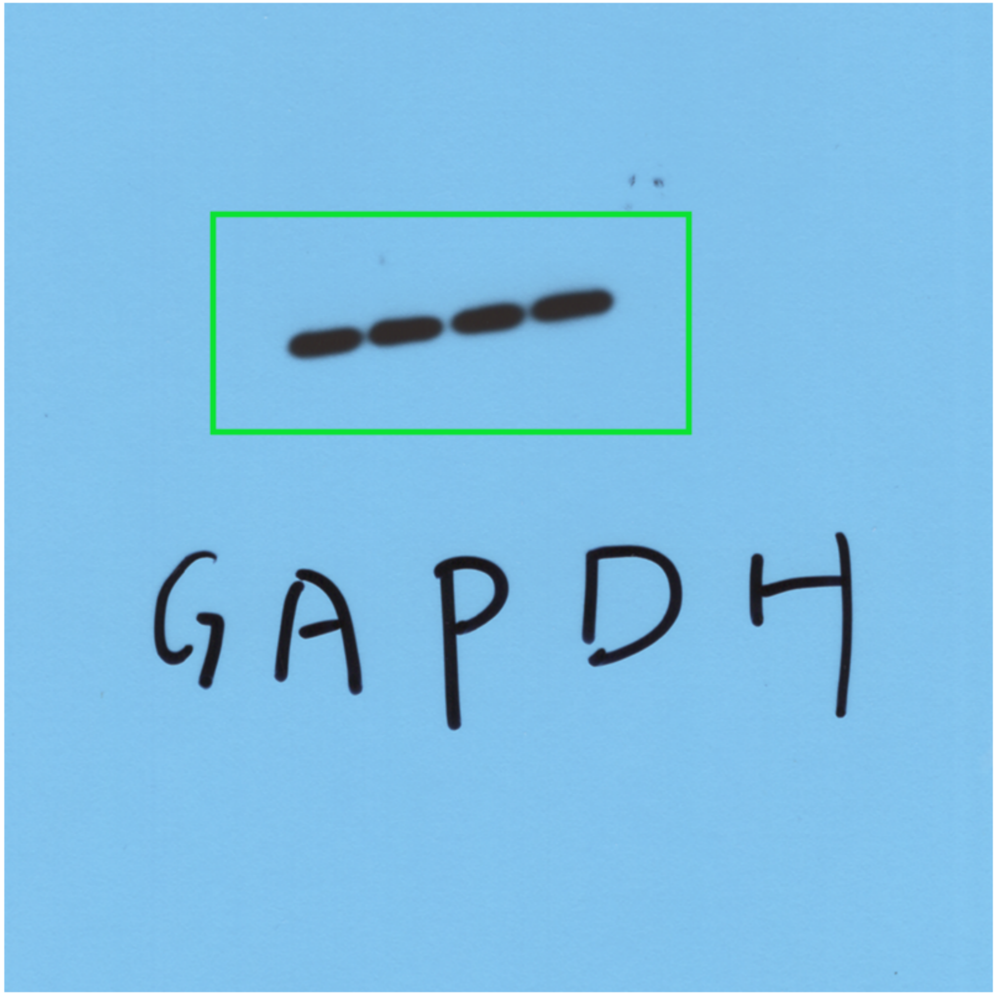
GAPDH

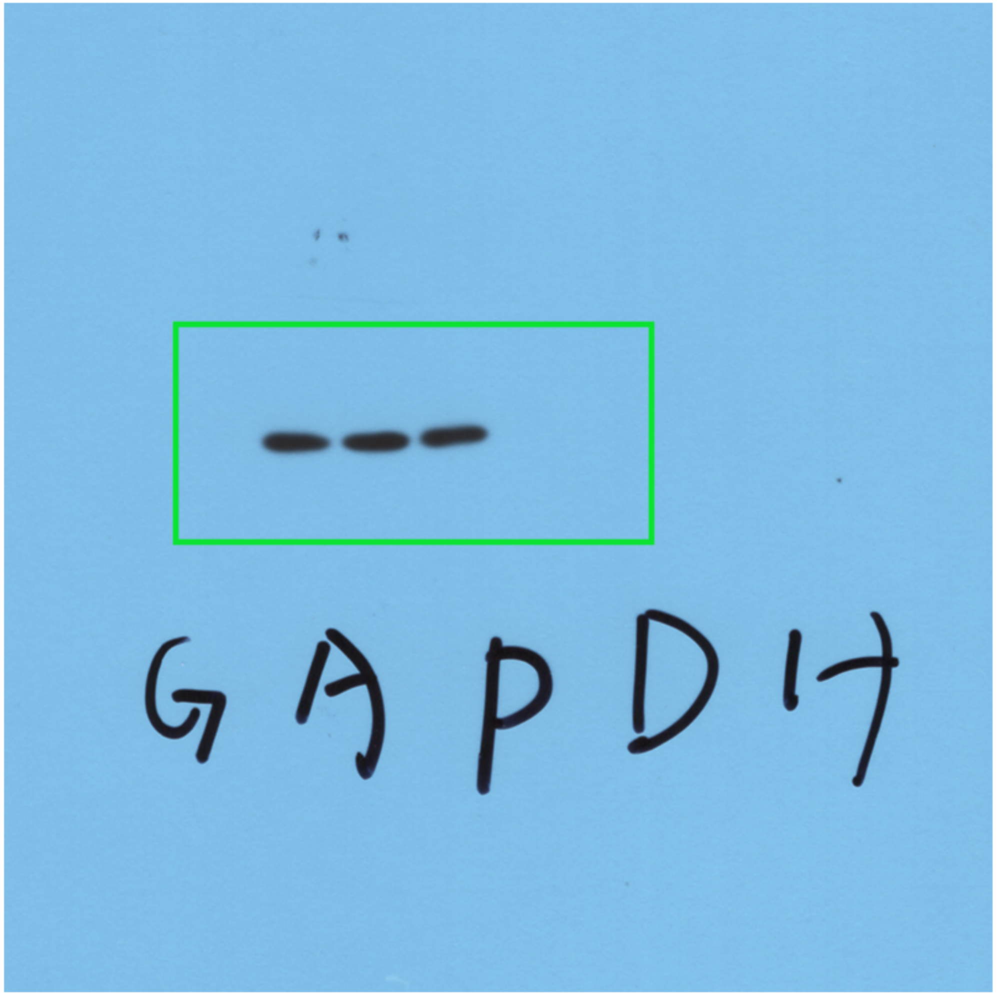
GAPDH

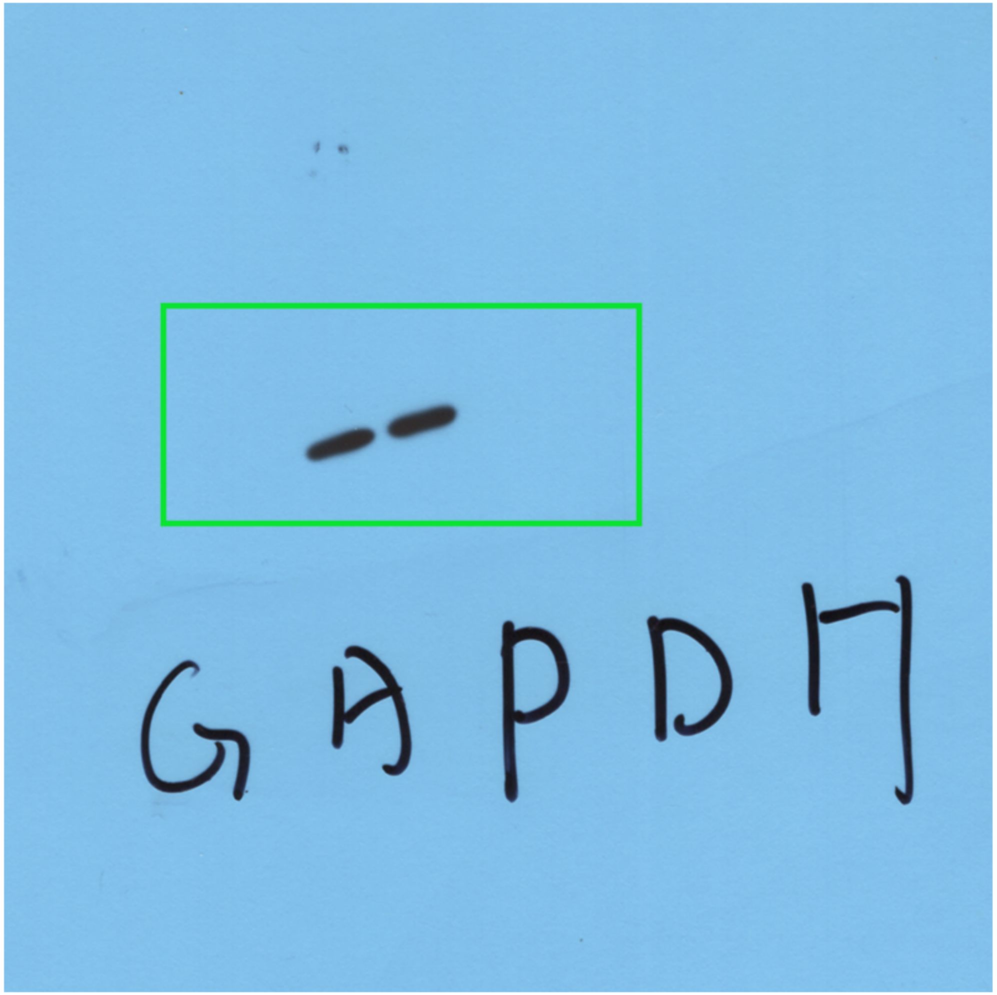
GAPDH

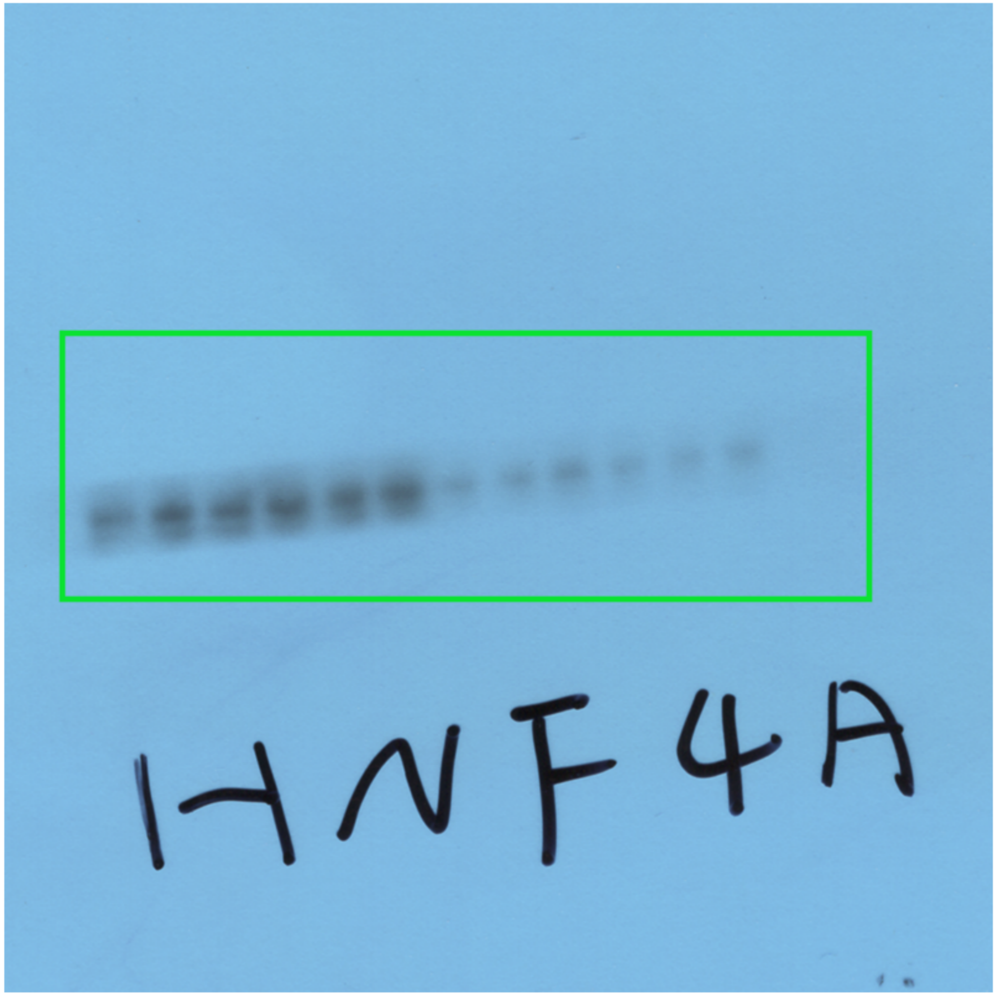
HNF4A

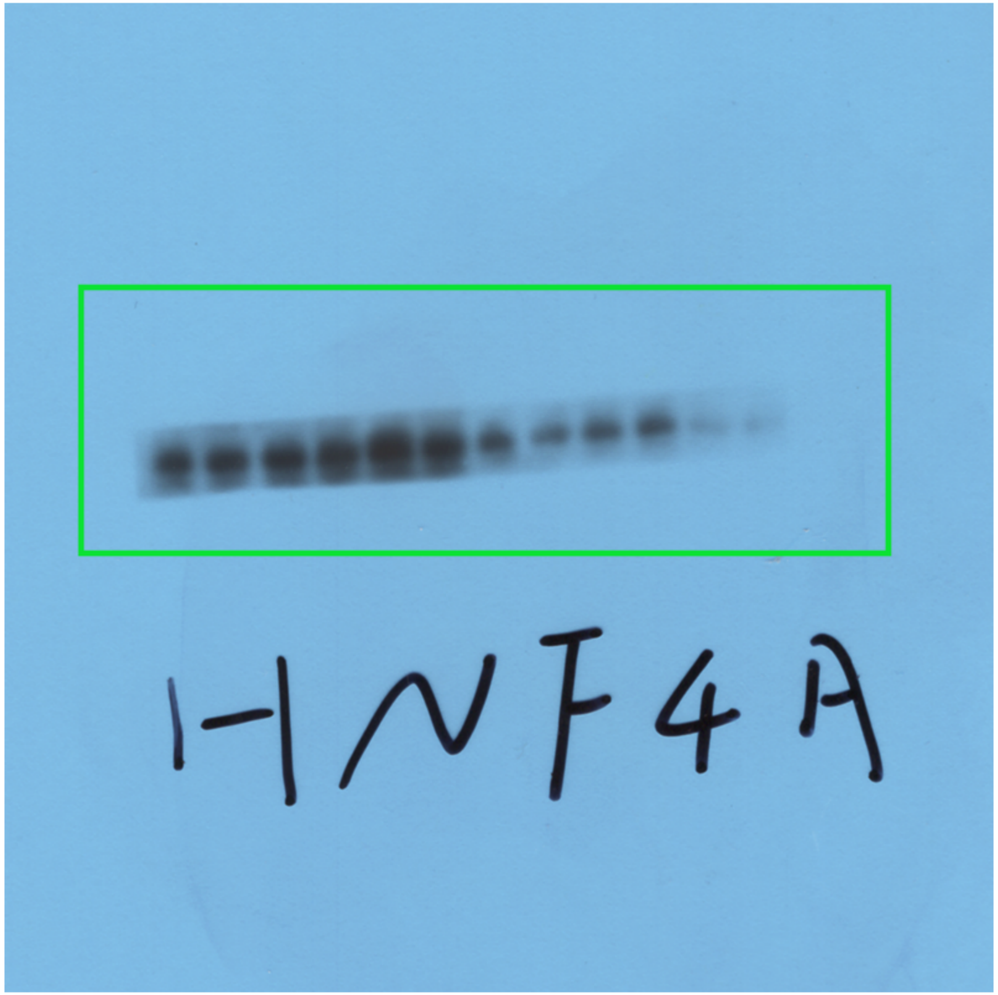
HNF4A

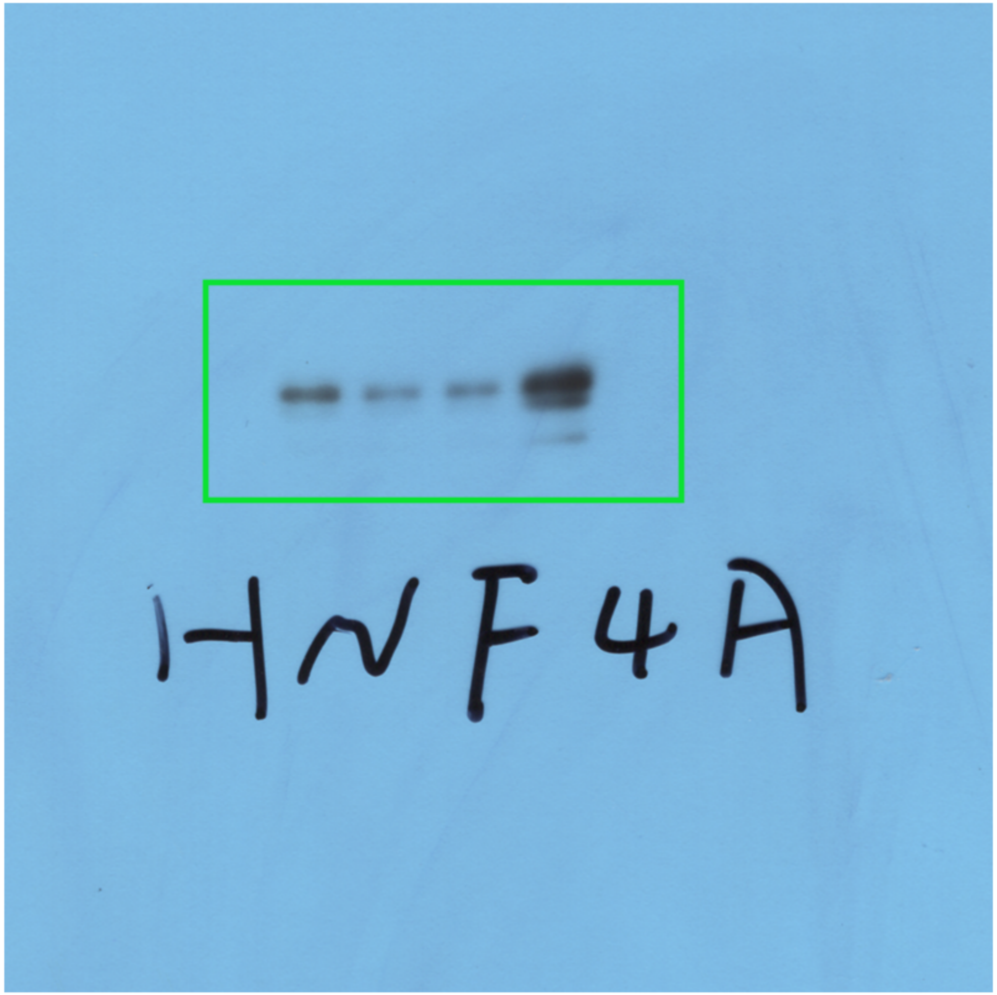
HNF4A

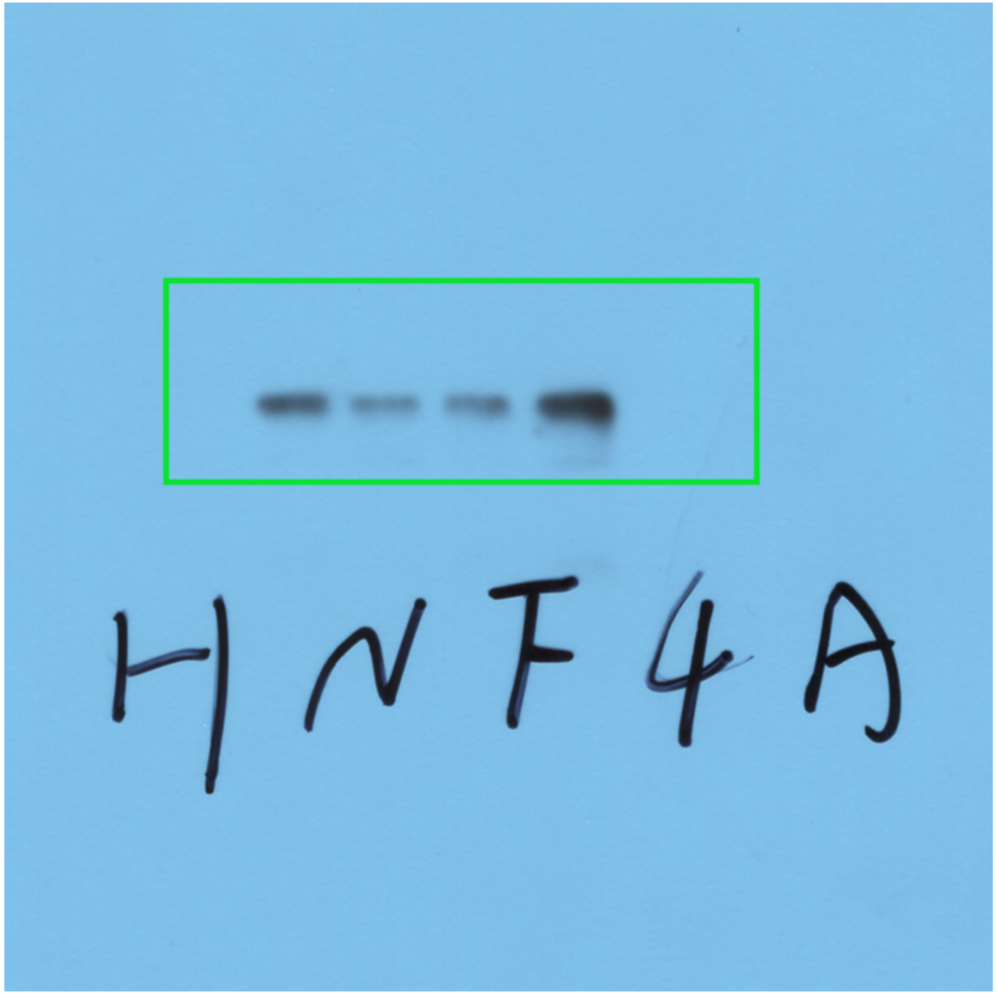
HNF4A

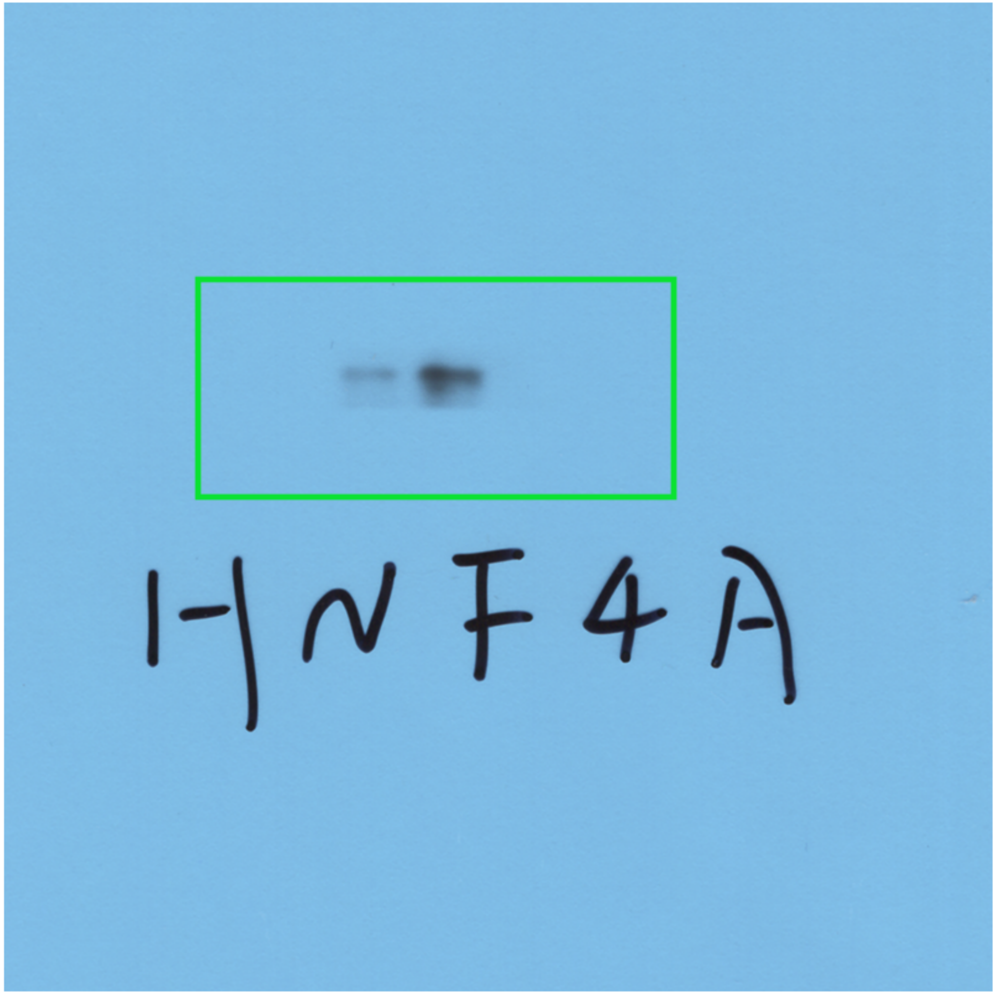
HNF4A

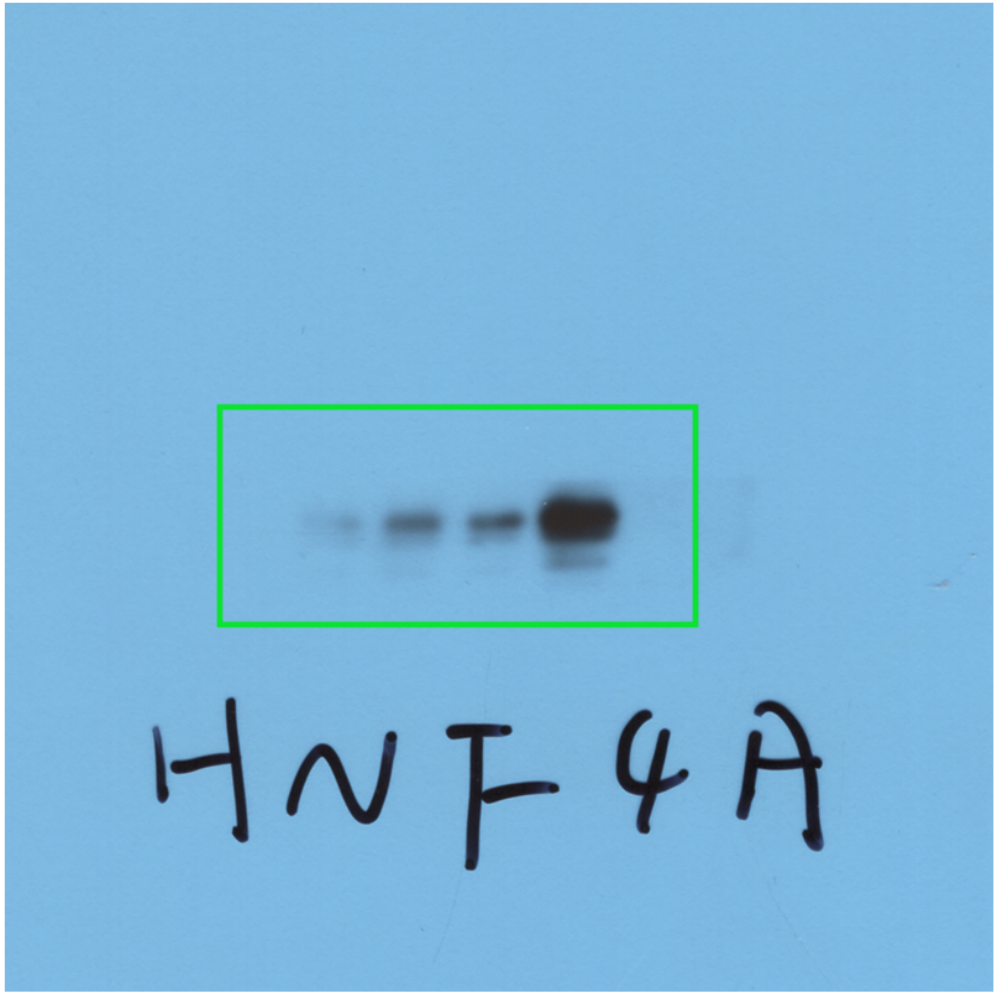
HNF4A

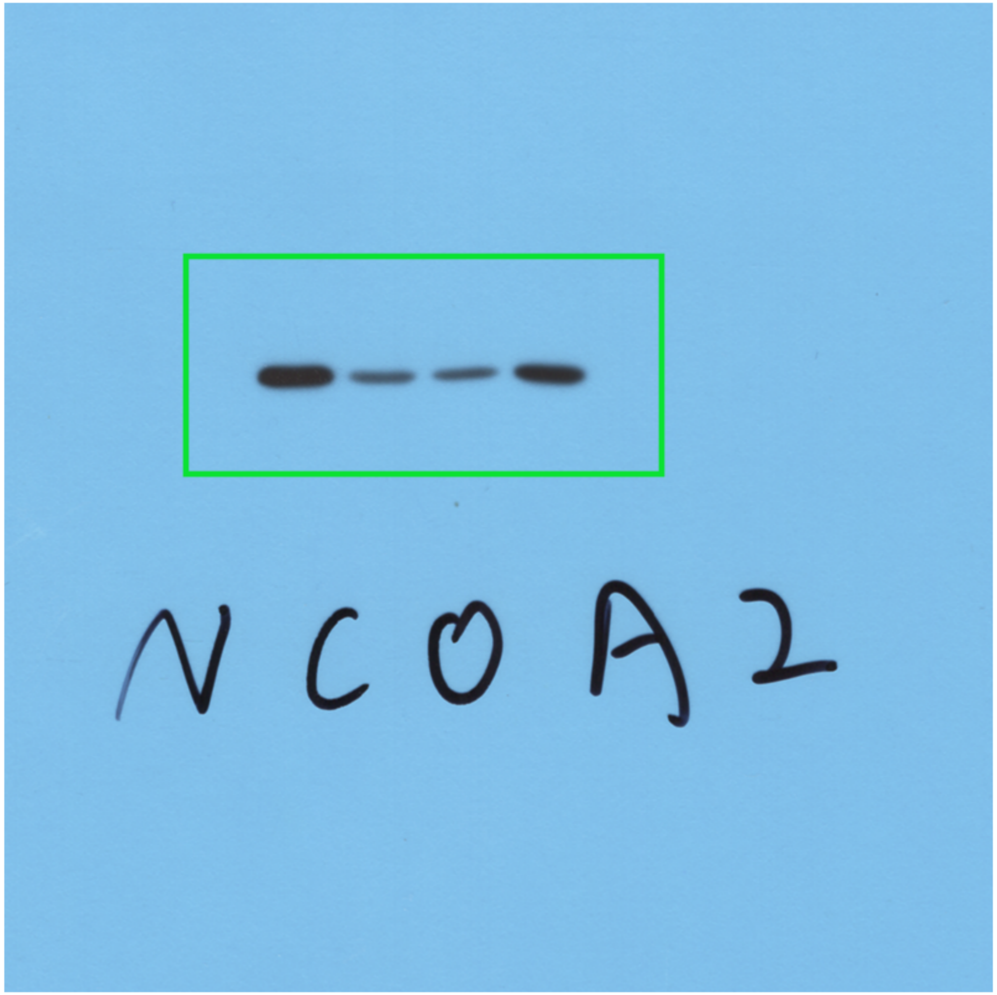
NCOA2

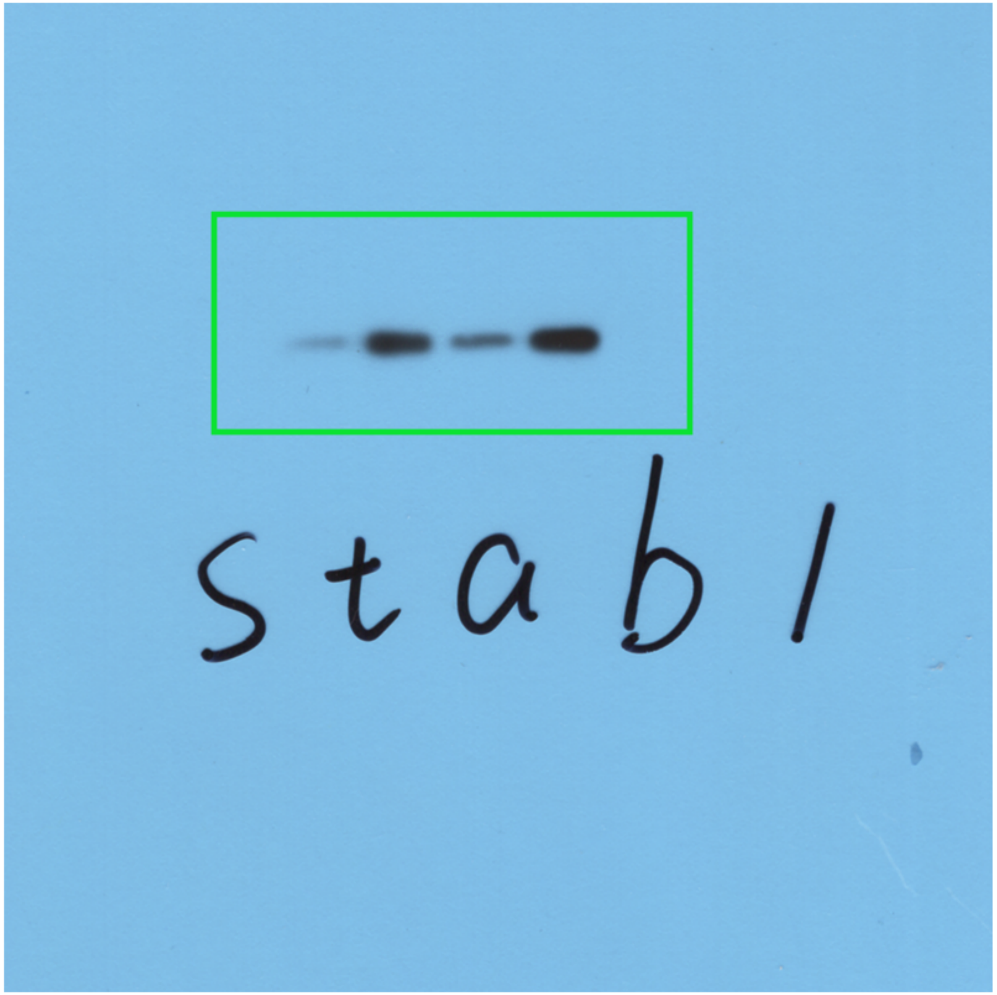
stab1

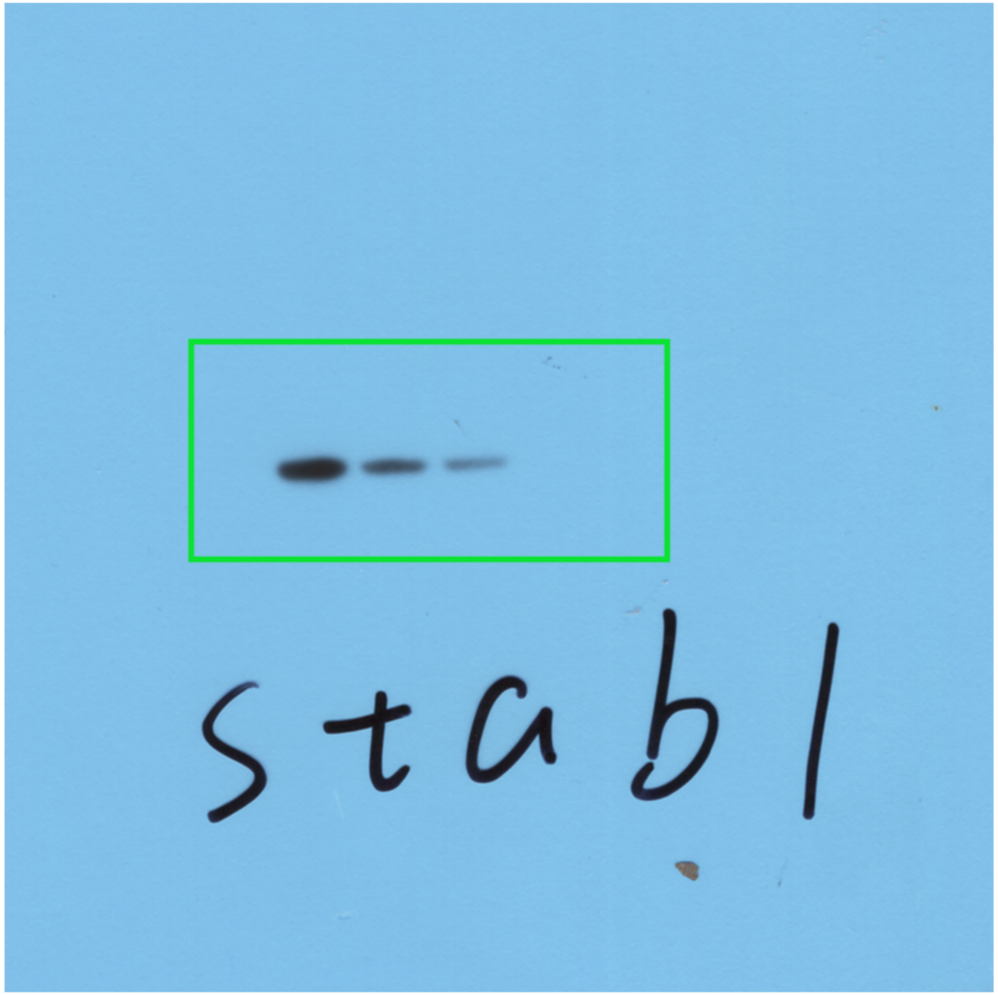
stab1

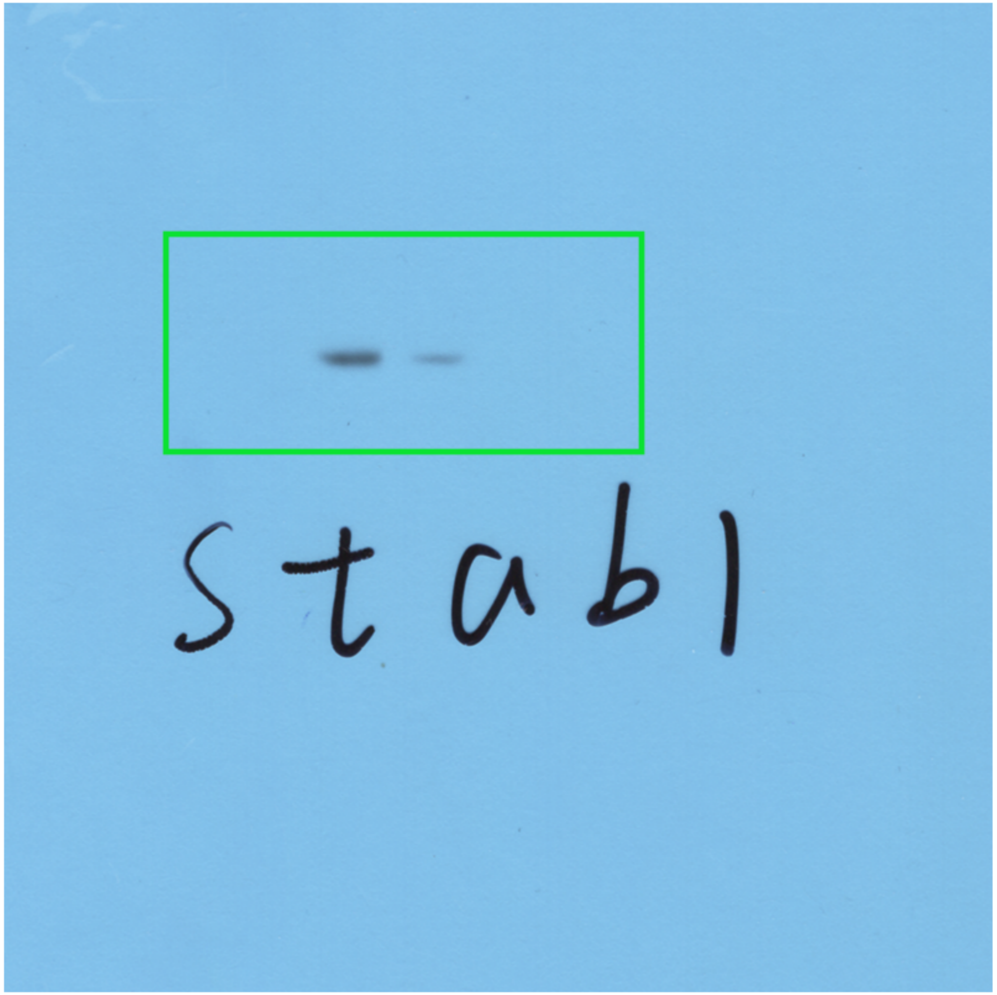
stab1
